# Supplementary material for: Navigating the shots: Parental willingness to immunize their children with COVID-19 vaccines in Saudi Arabia explored through a systematic review and meta-analysis
Source: PLoS One. 2025 Jan 27;20(1):e0317983. doi: 10.1371/journal.pone.0317983 (PMC11771943; doi:10.1371/journal.pone.0317983)
Supplement: S1 Table — (PDF) [file pone.0317983.s002.pdf]

S1 Table. Articles excluded from this SRMA and reasons for exclusion

Articles/Records removed before screening: Duplicate records (n = 204)

Articles/ Records excluded during screening by Title/Abstract (n = 304)

Serial	Article	Remarks
1	Alqahtani YS. Acceptability of the COVID-19 Vaccine among Adults in Saudi Arabia: A Cross-Sectional Study of the General Population in the Southern Region of Saudi Arabia. Vaccines (Basel). 2021 Dec 29;10(1):41.	
2	AlOmran HI, Al-Dosary AS, AlGhamdi FM, Alshahrani ZM, Altayar NS. Exploring parents' knowledge and attitudes towards the influenza vaccine in a rural community of Saudi Arabia. J Public Health Afr. 2022 May 24;13(1):2207.	
3	Alkhalifah JM, Al Seraihi A, Al-Tawfiq JA, Alshehri BF, Alhaluli AH, Alsulais NM, et al. Pattern of self-reported adverse events related to COVID-19 vaccines in Saudi Arabia: A nationwide study. Front Public Health. 2023 Feb 23;11:1043696.	
4	Alzeer AA, Alfantoukh LA, Theneyan A, Bin Eid F, Almangour TA, Alshememry AK, et al. The influence of demographics on influenza vaccine awareness and hesitancy among adults visiting educational hospital in Saudi Arabia. Saudi Pharm J. 2021 Feb;29(2):188-193.	
5	Alahmadi AM, Aljohani AH, Fadhloun RA, Almohammadi AS, Alharbi DF, Alrefai LS. The Effect of the COVID-19 Vaccine on the Menstrual Cycle Among Reproductive-Aged Females in Saudi Arabia. Cureus. 2022 Dec 13;14(12):e32473.	
6	Al-Hanawi MK, Alshareef N, El-Sokkary RH. Willingness to Receive COVID-19 Vaccination among Older Adults in Saudi Arabia: A Community-Based Survey. Vaccines (Basel). 2021 Oct 31;9(11):1257.	
7	Alabadi M, Alashoor T, Aldawood O, Qanbar Z, Aldawood Z. Exploring Critical Factors Associated with Completion of Childhood Immunisation in the Eastern Province of Saudi Arabia. Vaccines (Basel). 2022 Dec 14;10(12):2147.	
8	AlHefdhi HA, Mahmood SE, Alsaeedi MAI, Alwabel HHA, Alshahrani MS, Alshehri EY, et al. COVID-19 Vaccine Uptake and Hesitancy among Pregnant and Lactating Women in Saudi Arabia. Vaccines (Basel). 2023 Feb 5;11(2):361.	
9	Al-Mohaithef M, Padhi BK. Determinants of COVID-19 vaccine acceptance in Saudi Arabia: A web-based national survey. J Multidiscip Healthc. (2020) 13:1657–63.	
10	Lee HW, Leng CH, Chan TC. Determinants of personal vaccination hesitancy before and after the mid-2021 COVID-19 outbreak in Taiwan. PLoS One. 2022 Jul 29;17(7):e0270349.	
11	Abu-Farha R, Mukattash T, Itani R, Karout S, Khojah HMJ, Abed Al-Mahmood A, Alzoubi KH. Willingness of Middle Eastern public to receive COVID-19 vaccines. Saudi Pharm J. 2021 Jul;29(7):734-739.	
12	Alfageeh EI, Alshareef N, Angawi K, Alhazmi F, Chirwa GC. Acceptability of a COVID-19 Vaccine among the Saudi Population. Vaccines (Basel). 2021 Mar 5;9(3):226.	
13	Alobaidi S. Predictors of Intent to Receive the COVID-19 Vaccination Among the Population in the Kingdom of Saudi Arabia: A Survey Study. J Multidiscip Healthc. 2021 May 18;14:1119-1128.	
14	Alshahrani SM, Dehom S, Almutairi D, Alnasser BS, Alsaif B, Alabdrabalnabi AA, et al. Acceptability of COVID-19 vaccination in Saudi Arabia: A cross-sectional study using a web-based survey. Hum Vaccin Immunother. 2021 Oct 3;17(10):3338-3347.	
15	Baghdadi LR, Alghaihb SG, Abuhaimed AA, Alkelabi DM, Alqahtani RS. Healthcare Workers' Perspectives on the Upcoming COVID-19 Vaccine in Terms of Their Exposure to the Influenza Vaccine in Riyadh, Saudi Arabia: A Cross-Sectional Study. Vaccines (Basel). 2021 May 6;9(5):465.	
16	Abullais SS, Arora S, Al Shahrani M, Khan AA, Al Shahrani W, Mahmood SE, et al. Knowledge, perception, and acceptance toward the booster dose of COVID-19 vaccine among patients visiting dental clinics in Aseer region of KSA. Hum Vaccin Immunother. 2022 Nov 30;18(6):2095162.	
17	Temsah MH, Barry M, Aljamaan F, Alhuzaimi A, Al-Eyadhy A, Saddik B, et al. Adenovirus and RNA-based COVID-19 vaccines' perceptions and acceptance among healthcare workers in Saudi Arabia: a national survey. BMJ Open. 2021 Jun 21;11(6):e048586.	
18	Almaghasla D, Alsayari A, Wahab S, Motaal AA. Knowledge, attitudes and practices with regard to prophetic medicine during the COVID-19 pandemic in Saudi Arabia. Eur Rev Med Pharmacol Sci. 2023 Jan;27(1):352-358.	

**19**	Al-Qerem W, Jarab A, Hammad A, Alasmari F, Ling J, Alsajri AH, et al. Iraqi Parents' Knowledge, Attitudes, and Practices towards Vaccinating Their Children: A Cross-Sectional Study. Vaccines (Basel). 2022 May 22;10(5):820.	
**20**	Alshahrani SM, Alotaibi A, Almajed E, Alotaibi A, Alotaibi K, Albisher S. Pregnant and Breastfeeding Women's Attitudes and Fears Regarding COVID-19 Vaccination: A Nationwide Cross-Sectional Study in Saudi Arabia. Int J Womens Health. 2022 Nov 25;14:1629-1639.	
**21**	Tomietto M, Simonetti V, Comparcini D, Stefanizzi P, Cicolini G. A large cross-sectional survey of COVID-19 vaccination willingness amongst healthcare students and professionals: Reveals generational patterns. J Adv Nurs. 2022 Sep;78(9):2894-2903.	
**22**	Gallè F, Sabella EA, Roma P, Da Molin G, Diella G, Montagna MT, et al. Acceptance of COVID-19 Vaccination in the Elderly: A Cross-Sectional Study in Southern Italy. Vaccines (Basel). 2021 Oct 21;9(11):1222.	
**23**	Rellosa N. COVID-19 Vaccine Hesitancy and Refusal:: The Same But Different? Dela J Public Health. 2022 Mar 29;8(1):72-75.	
**24**	Al-Wutayd O, Al-Batanony M, Badr N, Abdelwanees S. Parents' Intentions and Associated Factors to Vaccinating Their Children Aged 12-17 Years with COVID-19 Vaccines: A Cross Sectional Study. Vaccines (Basel). 2022 Jun 8;10(6):912.	
**25**	Chutiyami M, Bello UM, Salihu D, Kolo MA, Alsharari AF, Sabo H, Bukar M, Shehu U, Adamu H, Alkali HI, et al. Subjective Reasons for COVID-19 Vaccine Hesitancy and Sociodemographic Predictors of Vaccination in Nigeria: An Online Survey. COVID. 2022; 2(10):1329-1340.	
**26**	Shahwan M, Suliman A, Abdulrahman Jairoun A, Alkhoujah S, Al-Hemyari SS, Al-Tamimi SK, et al. Prevalence, Knowledge and Potential Determinants of COVID-19 Vaccine Acceptability Among University Students in the United Arab Emirates: Findings and Implications. J Multidiscip Healthc. 2022 Jan 11;15:81-92.	
**27**	Latif R, Alali S, AlNujaidi R, Alotaibi L, Alghamdi N, Alblaies M. COVID-19: Risk Stratification of Healthcare Workers in the Eastern Province of Saudi Arabia and Their Knowledge, Attitude, and Fears. Cureus. 2021 Nov 17;13(11):e19652.	
**28**	Durmaz N, Suman M, Ersoy M, Örün E. Parents' Attitudes toward Childhood Vaccines and COVID-19 Vaccines in a Turkish Pediatric Outpatient Population. Vaccines (Basel). 2022 Nov 18;10(11):1958.	
**29**	Magadmi RM, Kamel FO. Beliefs and barriers associated with COVID-19 vaccination among the general population in Saudi Arabia. BMC Public Health. 2021 Jul 21;21(1):1438.	
**30**	Al-Mistarehi AH, Kheirallah KA, Yassin A, Alomari S, Aledrisi MK, Bani Ata EM, et al. Determinants of the willingness of the general population to get vaccinated against COVID-19 in a developing country. Clin Exp Vaccine Res. 2021 May;10(2):171-182.	
**31**	Alrabiaah AA, Alshaer AH, Estrella SMC, Inclan KAS, Aljammaz HA, Almoosa KM, et al. Effects of the Coronavirus disease 2019 pandemic on routine pediatric immunization coverage rates at the main University Hospital in Saudi Arabia. Saudi Med J. 2020 Nov;41(11):1197-1203.	
**32**	Alshareef N. COVID-19 Vaccine Acceptance and Associated Factors among Women in Saudi Arabia: A Cross-Sectional Study. Vaccines (Basel). 2022 Oct 31;10(11):1842.	
**33**	Alibrahim J, Awad A. COVID-19 Vaccine Hesitancy among the Public in Kuwait: A Cross-Sectional Survey. Int J Environ Res Public Health. 2021 Aug 22;18(16):8836.	
**34**	Ibrahim FM, Fadila DE, Elmawla DAEA. Older adults' acceptance of the COVID-19 vaccine: Application of the health belief model. Nurs Open. 2023 Oct;10(10):6989-7002.	
**35**	Khan AA, Abdullah M, Aliani R, Mohiuddin AF, Sultan F. COVID-19 vaccine hesitancy and attitudes in Pakistan: a cross-sectional phone survey of major urban cities. BMC Public Health. 2023 Jun 9;23(1):1112.	
**36**	Zakar R, Momina AU, Shahzad S, Hayee M, Shahzad R, Zakar MZ. COVID-19 Vaccination Hesitancy or Acceptance and Its Associated Factors: Findings from Post-Vaccination Cross-Sectional Survey from Punjab Pakistan. Int J Environ Res Public Health. 2022 Jan 24;19(3):1305.	
**37**	Shibani M, Alzabibi MA, Mouhandes AE, Alsuliman T, Mouki A, Ismail H, et al. COVID-19 vaccination acceptance among Syrian population: a nationwide cross-sectional study. BMC Public Health. 2021 Nov 18;21(1):2117.	
**38**	Mahmud I, Kabir R, Rahman MA, Alradie-Mohamed A, Vinnakota D, Al-Mohaimeed A. The Health Belief Model Predicts Intention to Receive the COVID-19 Vaccine in Saudi Arabia: Results from a Cross-Sectional Survey. Vaccines (Basel). 2021 Aug 5;9(8):864.	

**39**	Sowa P, Kiszkiel Ł, Laskowski PP, Alimowski M, Szczerbiński Ł, Paniczko M, et al. COVID-19 Vaccine Hesitancy in Poland-Multifactorial Impact Trajectories. Vaccines (Basel). 2021 Aug 7;9(8):876.	
**40**	Samannodi M. COVID-19 Vaccine Acceptability Among Women Who are Pregnant or Planning for Pregnancy in Saudi Arabia: A Cross-Sectional Study. Patient Prefer Adherence. 2021 Nov 23;15:2609-2618.	
**41**	Meo SA, Fahad Al-Jassir F, Al-Qahtani S, Albarrak R, Usmani AM, Klonoff DC. Effect of Pfizer/BioNTech and Oxford/AstraZeneca vaccines against COVID-19 morbidity and mortality in real-world settings at countrywide vaccination campaign in Saudi Arabia. Eur Rev Med Pharmacol Sci. 2021 Nov;25(22):7185-7191.	
**42**	Alamer E, Hakami F, Hamdi S, Alamer A, Awaf M, Darraj H, et al. Knowledge, Attitudes and Perception toward COVID-19 Vaccines among Adults in Jazan Province, Saudi Arabia. Vaccines (Basel). 2021 Nov 1;9(11):1259.	
**43**	Soleimanpour H, Sarbazi E, Esmaeili ED, Mehri A, Fam SG, Nikbakht HA, Saadati M, Sedighi S, Vali M, Azizi H. Predictors of receiving COVID-19 vaccine among adult population in Iran: an observational study. BMC Public Health. 2023 Mar 14;23(1):490.	
**44**	Ba MF, Faye A, Kane B, Diallo AI, Junot A, Gaye I, et al. Factors associated with COVID-19 vaccine hesitancy in Senegal: A mixed study. Hum Vaccin Immunother. 2022 Nov 30;18(5):2060020.	
**45**	Alshahrani NZ, Alshahrani SM, Farag S, Rashid H. Domestic Saudi Arabian Travellers' Understanding about COVID-19 and Its Vaccination. Vaccines (Basel). 2021 Aug 12;9(8):895.	
**46**	Al-Mansour K, Alyahya S, AbuGazalah F, Alabdulkareem K. Factors Affecting COVID-19 Vaccination among the General Population in Saudi Arabia. Healthcare (Basel). 2021 Sep 16;9(9):1218.	
**47**	Al-Mohaithef M, Padhi BK, Ennaceur S. Socio-Demographics Correlate of COVID-19 Vaccine Hesitancy During the Second Wave of COVID-19 Pandemic: A Cross-Sectional Web-Based Survey in Saudi Arabia. Front Public Health. 2021 Jun 24;9:698106.	
**48**	Almaghaslah D, Alsayari A, Kandasamy G, Vasudevan R. COVID-19 Vaccine Hesitancy among Young Adults in Saudi Arabia: A Cross-Sectional Web-Based Study. Vaccines (Basel). 2021 Apr 1;9(4):330.	
**49**	Al-Hanawi MK, Keetile M, Kadasah NA, Alshareef N, Qattan AMN, Alsharqi O. Side Effects and Perceptions of COVID-19 Vaccination in Saudi Arabia: A Cross-Sectional Study. Front Med (Lausanne). 2022 Jun 7;9:899517.	
**50**	Noushad M, Nassani MZ, Koppolu P, Alsalhani AB, Samran A, Alqerban A, et al. Predictors of COVID-19 Vaccine Intention among the Saudi Arabian Population: A Cross-Sectional Survey. Vaccines (Basel). 2021 Aug 12;9(8):892.	
**51**	Zahid HM, Alsayb MA. Assessing the Knowledge and Attitude toward COVID-19 Vaccination in Saudi Arabia. Int J Environ Res Public Health. 2021 Aug 2;18(15):8185.	
**52**	Jayakumar S, Ilango S, Kumar K S, Alassaf A, Aljabr A, Paramasivam A, et al. Contrasting Association Between COVID-19 Vaccine Hesitancy and Mental Health Status in India and Saudi Arabia-A Preliminary Evidence Collected During the Second Wave of COVID-19 Pandemic. Front Med (Lausanne). 2022 May 4;9:900026.	
**53**	Kurdee Z, Al-Shouli S, AlAfaleq N, Meo SA, Alshahrani A, Alshehri A, et al. Public Perception towards the COVID-19 Vaccine in Riyadh, Saudi Arabia. Vaccines (Basel). 2022 May 28;10(6):867.	
**54**	Okmi EA, Almohammadi E, Alaamri O, Alfawaz R, Alomari N, Alnughaymishi MAS, et al. Determinants of COVID-19 Vaccine Acceptance Among the General Adult Population in Saudi Arabia Based on the Health Belief Model: A Web-Based Cross-Sectional Study. Cureus. 2022 Aug 23;14(8):e28326.	
**55**	Faqihi E, Altwirki A, Mijlad W, Alzarie M, Alqumaizi F, Iqbal M, et al. Awareness, knowledge, attitudes, and practices before the second wave of the COVID-19 pandemic in Saudi Arabia. Eur Rev Med Pharmacol Sci. 2022 Jul;26(13):4926-4946.	
**56**	Almeshari M, Abanomy A, Alzamil Y, Alyahyawi A, Al-Thomali AW, Alshihri AA, et al. Public acceptance of COVID-19 vaccination among residents of Saudi Arabia: a cross-sectional online study. BMJ Open. 2022 Oct 31;12(10):e058180.	
**57**	Habib SS, Alamri MS, Alkhedr MM, Alkhorijah MA, Jabaan RD, Alanzi MK. Knowledge and Attitudes of Medical Students toward COVID-19 Vaccine in Saudi Arabia. Vaccines (Basel). 2022 Mar 31;10(4):541.	
**58**	Nour MO, Natto HA. COVID-19 vaccination acceptance and trust among adults in Makkah, Saudi Arabia: a cross-sectional study. J Egypt Public Health Assoc. 2022 Sep 26;97(1):17.	

**59**	Al-Ghuraibi M, Dighriri IM, Elrggal ME, Obaid NA. The socio-cultural factors behind the Saudi attitude toward COVID-19 vaccination: A survey-based study. Front Public Health. 2023 Jan 9;10:1026252.	
**60**	El Hassan ELW, Abu Alhommos AK, Aliadhy D, Alsalman S, Alnafaa O, Mohamed A. Public Knowledge, Beliefs and Attitudes toward the COVID-19 Vaccine in Saudi Arabia: A Cross-Sectional Study. Healthcare (Basel). 2022 May 5;10(5):853.	
**61**	Vellappally S, Naik S, Alsadon O, Al-Kheraif AA, Alayadi H, Alsiwat AJ, et al. Perception of COVID-19 Booster Dose Vaccine among Healthcare Workers in India and Saudi Arabia. Int J Environ Res Public Health. 2022 Jul 22;19(15):8942.	
**62**	Toro-Ascuy D, Cifuentes-Muñoz N, Avaria A, Pereira-Montecinos C, Cruzat G, Peralta-Arancibia K, et al. Factors Influencing the Acceptance of COVID-19 Vaccines in a Country with a High Vaccination Rate. Vaccines (Basel). 2022 Apr 25;10(5):681.	
**63**	Parsons Leigh J, Halperin D, Mizen SJ, FitzGerald EA, Moss SJ, Fiest KM, et al. Exploring the impact of media and information on self-reported intentions to vaccinate against COVID-19: A qualitative interview-based study. Hum Vaccin Immunother. 2022 Nov 30;18(5):2048623.	
**64**	Liao Q, Cowling BJ, Xiao J, Yuan J, Dong M, Ni MY, et al. Priming with social benefit information of vaccination to increase acceptance of COVID-19 vaccines. Vaccine. 2022 Feb 16;40(8):1074-1081.	
**65**	Ayele AD, Ayenew NT, Tenaw LA, Kassa BG, Yehuala ED, Aychew EW, et al. Acceptance of COVID-19 vaccine and associated factors among health professionals working in Hospitals of South Gondar Zone, Northwest Ethiopia. Hum Vaccin Immunother. 2021 Dec 2;17(12):4925-4933.	
**66**	Hanna P, Issa A, Noujeim Z, Hleyhel M, Saleh N. Assessment of COVID-19 vaccines acceptance in the Lebanese population: a national cross-sectional study. J Pharm Policy Pract. 2022 Jan 11;15(1):5.	
**67**	Marzo RR, Ahmad A, Islam MS, Essar MY, Heidler P, King I, et al. Perceived COVID-19 vaccine effectiveness, acceptance, and drivers of vaccination decision-making among the general adult population: A global survey of 20 countries. PLoS Negl Trop Dis. 2022 Jan 28;16(1):e0010103.	
**68**	Akbas Gunes N. Parents' Perspectives about Vaccine Hesitancies and Vaccine Rejection, in the West of Turkey. J Pediatr Nurs. 2020 Jul-Aug;53:e186-e194.	
**69**	Gjini E, Moramarco S, Carestia MC, Cenko F, Ylli A, Mehmeti I, et al. Parents' and caregivers' role toward childhood vaccination in Albania: assessment of predictors of vaccine hesitancy. Ann Ig. 2023 Jan-Feb;35(1):75-83.	
**70**	Dubé È, Ward JK, Verger P, MacDonald NE. Vaccine Hesitancy, Acceptance, and Anti-Vaccination: Trends and Future Prospects for Public Health. Annu Rev Public Health. 2021 Apr 1;42:175-191.	
**71**	Alabadi M, Pitt V, Aldawood Z. A Qualitative Analysis of Social-Ecological Factors Shaping Childhood Immunisation Hesitancy and Delay in the Eastern Province of Saudi Arabia. Vaccines (Basel). 2023 Aug 22;11(9):1400.	
**72**	Alaamri O, Okmi EA, Suliman Y. Vaccine Hesitancy in Saudi Arabia: A Cross-Sectional Study. Trop Med Infect Dis. 2022 Apr 12;7(4):60.	
**73**	Wagner AL, Shotwell AR, Boulton ML, Carlson BF, Mathew JL. Demographics of Vaccine Hesitancy in Chandigarh, India. Front Med (Lausanne). 2021 Jan 15;7:585579.	
**74**	Szilagyi PG, Albertin CS, Gurfinkel D, Saville AW, Vangala S, Rice JD, et al. Prevalence and characteristics of HPV vaccine hesitancy among parents of adolescents across the US. Vaccine. 2020 Aug 27;38(38):6027-6037.	
**75**	Kempe A, Saville AW, Albertin C, Zimet G, Breck A, Helmkamp L, et al. Parental Hesitancy About Routine Childhood and Influenza Vaccinations: A National Survey. Pediatrics. 2020 Jul;146(1):e20193852.	
**76**	Yufika A, Wagner AL, Nawawi Y, Wahyuniati N, Anwar S, Yusri F, et al. Parents' hesitancy towards vaccination in Indonesia: A cross-sectional study in Indonesia. Vaccine. 2020 Mar 4;38(11):2592-2599.	
**77**	Novilla MLB, Goates MC, Redelfs AH, Quenzer M, Novilla LKB, Leffler T, et al. Why Parents Say No to Having Their Children Vaccinated against Measles: A Systematic Review of the Social Determinants of Parental Perceptions on MMR Vaccine Hesitancy. Vaccines (Basel). 2023 May 2;11(5):926.	
**78**	Darraj MA, Al-Mekhlafi HM. Prospective Evaluation of Side-Effects Following the First Dose of Oxford/AstraZeneca COVID-19 Vaccine among Healthcare Workers in Saudi Arabia. Vaccines (Basel). 2022 Jan 30;10(2):223.	
**79**	Migriño J Jr, Gayados B, Birol KRJ, De Jesus L, Lopez CW, Mercado WC, et al. Factors affecting vaccine hesitancy among families with children 2 years old and younger in two urban communities in Manila, Philippines. Western Pac Surveill Response J. 2020 Jun 30;11(2):20-26.	

**80**	Thabit AK, Badr AF, Jad L, Jose J, Kaae S, Jacobsen R, et al. Views and perceptions of the public toward COVID-19 vaccine in Saudi Arabia. Saudi Pharm J. 2023 Jun;31(6):942-947.	
**81**	Gravelle TB, Phillips JB, Reifler J, Scotto TJ. Estimating the size of "anti-vax" and vaccine hesitant populations in the US, UK, and Canada: comparative latent class modeling of vaccine attitudes. Hum Vaccin Immunother. 2022 Dec 31;18(1):2008214.	
**82**	Ledda C, Costantino C, Liberti G, Rapisarda V. The Italian Version of the Adult Vaccine Hesitancy Scale (aVHS) for the Working-Age Population: Cross-Cultural Adaptation, Reliability, and Validity. Vaccines (Basel). 2022 Jan 31;10(2):224.	
**83**	Marshall S, Moore AC, Sahm LJ, Fleming A. Parent Attitudes about Childhood Vaccines: Point Prevalence Survey of Vaccine Hesitancy in an Irish Population. Pharmacy (Basel). 2021 Nov 23;9(4):188.	
**84**	Wang Q, Xiu S, Yang L, Han Y, Huang J, Cui T, et al. Delays in routine childhood vaccinations and their relationship with parental vaccine hesitancy: a cross-sectional study in Wuxi, China. Expert Rev Vaccines. 2022 Jan;21(1):135-143.	
**85**	Bednarczyk RA, Dew MA, Hart TA, Freedland KE, Kaufmann PG. Introduction to the special issue on vaccine hesitancy and refusal. Health Psychol. 2023 Aug;42(8):511-515.	
**86**	Biella M, Orrù G, Ciacchini R, Conversano C, Marazziti D, Gemignani A. Anti-Vaccination Attitude and Vaccination Intentions Against Covid-19: A Retrospective Cross-Sectional Study Investigating the Role of Media Consumption. Clin Neuropsychiatry. 2023 Aug;20(4):252-263.	
**87**	Huang Y, Wu Y, Dai Z, Xiao W, Wang H, Si M, et al. Psychometric validation of a Chinese version of COVID-19 vaccine hesitancy scale: a cross-sectional study. BMC Infect Dis. 2022 Oct 1;22(1):765.	
**88**	Shen AK, Grundmeier RW, Michel JJ. Trends in Vaccine Refusal and Acceptance Using Electronic Health Records from a Large Pediatric Hospital Network, 2013-2020: Strategies for Change. Vaccines (Basel). 2022 Oct 10;10(10):1688.	
**89**	Hadjipanayis A, van Esso D, Del Torso S, Dornbusch HJ, Michailidou K, Minicuci N, et al. Vaccine confidence among parents: Large scale study in eighteen European countries. Vaccine. 2020 Feb 5;38(6):1505-1512.	
**90**	Harada T, Watanabe T. Changes in Vaccine Hesitancy in Japan across Five Months during the COVID-19 Pandemic and Its Related Factors. Vaccines (Basel). 2021 Dec 26;10(1):25.	
**91**	Han Y, Wang Q, Zhao S, Wang J, Dong S, Cui T, et al. Parental category B vaccine hesitancy and associated factors in China: an online cross-sectional survey. Expert Rev Vaccines. 2022 Jan;21(1):145-153.	
**92**	Cataldi JR, O'Leary ST. Parental vaccine hesitancy: scope, causes, and potential responses. Curr Opin Infect Dis. 2021 Oct 1;34(5):519-526.	
**93**	Chung-Delgado K, Valdivia Venero JE, Vu TM. Vaccine Hesitancy: Characteristics of the Refusal of Childhood Vaccination in a Peruvian Population. Cureus. 2021 Mar 25;13(3):e14105.	
**94**	Adamu AA, Essoh TA, Adeyanju GC, Jalo RI, Saleh Y, Aplogan A, et al. Drivers of hesitancy towards recommended childhood vaccines in African settings: a scoping review of literature from Kenya, Malawi and Ethiopia. Expert Rev Vaccines. 2021 May;20(5):611-621.	
**95**	Fonseca IC, Pereira AI, Barros L. Portuguese parental beliefs and attitudes towards vaccination. Health Psychol Behav Med. 2021 May 6;9(1):422-435.	
**96**	Vasudevan L, Baumgartner JN, Moses S, Ngadaya E, Mfinanga SG, Ostermann J. Parental concerns and uptake of childhood vaccines in rural Tanzania - a mixed methods study. BMC Public Health. 2020 Oct 20;20(1):1573.	
**97**	Hamadah RE, Hussain AN, Alsoghayer NA, Alkhenizan ZA, Alajlan HA, Alkhenizan AH. Attitude of parents towards seasonal influenza vaccination for children in Saudi Arabia. J Family Med Prim Care. 2021 Feb;10(2):904-909.	
**98**	Nassani MZ, Noushad M, Rastam S, Hussain M, Alsalhani AB, Al-Saqqaf IS, et al. Determinants of COVID-19 Vaccine Acceptance among Dental Professionals: A Multi-Country Survey. Vaccines (Basel). 2022 Sep 26;10(10):1614.	
**99**	Elizondo-Alzola U, G Carrasco M, Pinós L, Picchio CA, Rius C, Diez E. Vaccine hesitancy among paediatric nurses: Prevalence and associated factors. PLoS One. 2021 May 19;16(5):e0251735.	
**100**	Sheikh NS, Touseef M, Sultan R, Cheema KH, Cheema SS, Sarwar A, et al. Understanding COVID-19 vaccine hesitancy in Pakistan: The paradigm of confidence, convenience, and complacency; A cross-sectional study. PLoS One. 2023 Aug 16;18(8):e0289678.	

**101**	Batool R, Yousafzai MT, Qureshi S, Muhammad S, Qazi I, Sadaf T, et al. Parental acceptance of typhoid conjugate vaccine for children aged 6 months to 15 years in an outbreak setting of Lyari Town Karachi, Pakistan. Vaccine. 2023 Aug 23;41(37):5376-5382.	
**102**	Luk TT, Lui JHT, Wang MP. Efficacy, Usability, and Acceptability of a Chatbot for Promoting COVID-19 Vaccination in Unvaccinated or Booster-Hesitant Young Adults: Pre-Post Pilot Study. J Med Internet Res. 2022 Oct 4;24(10):e39063.	
**103**	Alnumair A, Almulifi A. Perceptions toward childhood vaccinations (side effects vs. benefits) among the parents living in Hail, Saudi Arabia. J Family Med Prim Care. 2022 Oct;11(10):6285-6290.	
**104**	Butter S, McGlinchey E, Berry E, Armour C. Psychological, social, and situational factors associated with COVID-19 vaccination intentions: A study of UK key workers and non-key workers. Br J Health Psychol. 2022 Feb;27(1):13-29.	
**105**	Deml MJ, Buhl A, Notter J, Kliem P, Huber BM, Pfeiffer C, et al. 'Problem patients and physicians' failures': What it means for doctors to counsel vaccine hesitant patients in Switzerland. Soc Sci Med. 2020 Jun;255:112946.	
**106**	Kongo E, Shpati K, Dama A, Ymeraj S, Murati E, Veliaj U, et al. Determinant Factors of Voluntary or Mandatory Vaccination against COVID-19: A Survey Study among Students at Albanian University. Vaccines (Basel). 2023 Jul 7;11(7):1215.	
**107**	Inam A, Mushtaq A, Zaman S, Wasif S, Noor M, Khan HA. Vaccine hesitancy and post-vaccination adherence to safety measures: A mixed-method study. Front Public Health. 2023 Mar 31;11:1072740.	
**108**	Grignolio Corsini A, Zagarella RM, Adamo M, Caporale C. From COVID-19 vaccine candidates to compulsory vaccination: The attitudes of Italian citizens in the key 7-month of vaccination campaign. Vaccine. 2023 Apr 6;41(15):2582-2588.	
**109**	Hammoud H, Albayat SS, Mundodan J, Alateeg S, Adli N, Sabir D, et al. Development and validation of a multi-dimensional COVID-19 vaccine hesitancy questionnaire. Vaccine X. 2023 Aug;14:100286.	
**110**	Huang Y, Zhang L, Fu J, Wu Y, Wang H, Xiao W, et al. COVID-19 Vaccine Hesitancy Among Patients Recovered From COVID-19 Infection in Wuhan, China: Cross-Sectional Questionnaire Study. JMIR Public Health Surveill. 2023 Jul 3;9:e42958.	
**111**	Karagöz Özen DS, Karagöz Kiraz A, Yurt ÖF, Kiliç IZ, Demirağ MD. COVID-19 Vaccination Rates and Factors Affecting Vaccine Hesitancy among Pregnant Women during the Pandemic Period in Turkey: A Single-Center Experience. Vaccines (Basel). 2022 Nov 11;10(11):1910.	
**112**	Salman M, Mallhi TH, Tanveer N, Shehzadi N, Khan HM, Ul Mustafa Z, et al. Evaluation of Conspiracy Beliefs, Vaccine Hesitancy, and Willingness to Pay towards COVID-19 Vaccines in Six Countries from Asian and African Regions: A Large Multinational Analysis. Vaccines (Basel). 2022 Nov 4;10(11):1866.	
**113**	Lee SK, Sun J, Jang S, Connelly S. Misinformation of COVID-19 vaccines and vaccine hesitancy. Sci Rep. 2022 Aug 11;12(1):13681.	
**114**	Hou Z, Guo J, Lai X, Zhang H, Wang J, Hu S, et al. Influenza vaccination hesitancy and its determinants among elderly in China: A national cross-sectional study. Vaccine. 2022 Aug 5;40(33):4806-4815.	
**115**	Bonner KE, Ssekyanzi H, Sicsic J, Mueller JE, Toomey T, Ulrich AK, et al. What drives willingness to receive a new vaccine that prevents an emerging infectious disease? A discrete choice experiment among university students in Uganda. PLoS One. 2022 May 19;17(5):e0268063.	
**116**	Witus LS, Larson E. A randomized controlled trial of a video intervention shows evidence of increasing COVID-19 vaccination intention. PLoS One. 2022 May 19;17(5):e0267580.	
**117**	Kumar N, Corpus I, Hans M, Harle N, Yang N, McDonald C, et al. COVID-19 vaccine perceptions in the initial phases of US vaccine roll-out: an observational study on reddit. BMC Public Health. 2022 Mar 7;22(1):446.	
**118**	Allington D, McAndrew S, Duffy B, Moxham-Hall V. Trust and experiences of National Health Service healthcare do not fully explain demographic disparities in coronavirus vaccination uptake in the UK: a cross-sectional study. BMJ Open. 2022 Mar 18;12(3):e053827.	
**119**	Boness CL, Nelson M, Douaihy AB. Motivational Interviewing Strategies for Addressing COVID-19 Vaccine Hesitancy. J Am Board Fam Med. 2022 Mar-Apr;35(2):420-426.	
**120**	de Vries H, Verputten W, Preissner C, Kok G. COVID-19 Vaccine Hesitancy: The Role of Information Sources and Beliefs in Dutch Adults. Int J Environ Res Public Health. 2022 Mar 9;19(6):3205.	

**121**	Narapureddy BR, Muzammil K, Alshahrani MY, Alkhathami AG, Alsabaani A, AlShahrani AM, et al. COVID-19 Vaccine Acceptance: Beliefs and Barriers Associated with Vaccination Among the Residents of KSA. J Multidiscip Healthc. 2021 Nov 24;14:3243-3252.	
**122**	Gerretsen P, Kim J, Quilty L, Wells S, Brown EE, Agic B, et al. Vaccine Hesitancy Is a Barrier to Achieving Equitable Herd Immunity Among Racial Minorities. Front Med (Lausanne). 2021 Nov 24;8:668299.	
**123**	Hossain MB, Alam MZ, Islam MS, Sultan S, Faysal MM, Rima S, et al. Health Belief Model, Theory of Planned Behavior, or Psychological Antecedents: What Predicts COVID-19 Vaccine Hesitancy Better Among the Bangladeshi Adults? Front Public Health. 2021 Aug 16;9:711066.	
**124**	Nair AT, Nayar KR, Koya SF, Abraham M, Lordson J, Grace C, et al. Social media, vaccine hesitancy and trust deficit in immunization programs: a qualitative enquiry in Malappuram District of Kerala, India. Health Res Policy Syst. 2021 Aug 11;19(Suppl 2):56.	
**125**	Teasdale CA, Borrell LN, Shen Y, Kimball S, Rinke ML, Fleary SA, et al. Parental plans to vaccinate children for COVID-19 in New York city. Vaccine. 2021 Aug 23;39(36):5082-5086.	
**126**	Yilmaz S, Çolak FÜ, Yilmaz E, Ak R, Hökenek NM, Altıntaş MM. Vaccine Hesitancy of Health-Care Workers: Another Challenge in the Fight Against COVID-19 in Istanbul. Disaster Med Public Health Prep. 2022 Jun;16(3):1134-1140.	
**127**	Ugale JL, Spielvogle H, Spina C, Perreira C, Katz B, Pahud B, et al. "It's Like 1998 Again": Why Parents Still Refuse and Delay Vaccines. Glob Pediatr Health. 2021 Aug 27;8:2333794X211042331.	
**128**	Yörük S, Güler D. Factors associated with pediatric vaccine hesitancy of parents: a cross-sectional study in Turkey. Hum Vaccin Immunother. 2021 Nov 2;17(11):4505-4511.	
**129**	Caso D, Capasso M, Fabbricatore R, Conner M. Understanding the psychosocial determinants of Italian parents' intentions not to vaccinate their children: an extended theory of planned behaviour model. Psychol Health. 2022 Sep;37(9):1111-1131.	
**130**	Tsimtsiou Z, Tsiligianni I, Papaioannou A, Gougourelas D, Kolokas K, Gkizlis V, et al. Understanding what people think and feel about adult vaccinations and the associated barriers in Greece: Development and validation of the attitude towards adult vaccination (ATAVAC) scale. Health Soc Care Community. 2021 May;29(3):818-828.	
**131**	Goldman RD, Hart RJ, Bone JN, Seiler M, Olson PG, Keitel K, et al. Willingness to vaccinate children against COVID-19 declined during the pandemic. Vaccine. 2023 Apr 6;41(15):2495-2502.	
**132**	Goldman RD, Krupik D, Ali S, Mater A, Hall JE, Bone JN, et al. Caregiver Willingness to Vaccinate Their Children against COVID-19 after Adult Vaccine Approval. Int J Environ Res Public Health. 2021 Sep 28;18(19):10224.	
**133**	Baysson H, Pullen N, De Mestral C, Semaani C, Pennacchio F, Zaballa ME, et al. Parental willingness to have children vaccinated against COVID-19 in Geneva, Switzerland: a cross-sectional population-based study. Swiss Med Wkly. 2023 Apr 3;153(4):40049.	
**134**	Goldman RD, Yan TD, Seiler M, Parra Cotanda C, Brown JC, et al. Caregiver willingness to vaccinate their children against COVID-19: Cross sectional survey. Vaccine. 2020 Nov 10;38(48):7668-7673.	
**135**	Goldman RD, Ceballo R; International COVID-19 Parental Attitude Study (COVIPAS) Group. Parental gender differences in attitudes and willingness to vaccinate against COVID-19. J Paediatr Child Health. 2022 Jun;58(6):1016-1021.	
**136**	Goruntla N, Ayisha MU, Sreeram M. Predictors of Parents' Willingness to Vaccinate Their Children Against COVID-19 in India: A Web-Based Cross-Sectional Survey. Health Serv Res Manag Epidemiol. 2023 May 16;10:23333928231175798.	
**137**	Tung TH, Lin XQ, Chen Y, Wu H, Zhang MX, Zhu JS. Why do parents willingness-to-pay to vaccinate their children against COVID-19? A real-world evidence in Taizhou, China. Hum Vaccin Immunother. 2022 Dec 31;18(1):1-9.	
**138**	Salawati E, Alwafi H, Samannodi M, Minshawi F, Gari A, Abualnaja S, et al. Parents' Willingness to Vaccinate Their Children Against Seasonal Influenza After the COVID-19 Pandemic in Saudi Arabia: A Retrospective Cross-Sectional Survey. Patient Prefer Adherence. 2021 Dec 16;15:2821-2835.	
**139**	Miraglia Del Giudice G, Della Polla G, Postiglione M, Angelillo IF. Willingness and hesitancy of parents to vaccinate against COVID-19 their children ages 6 months to 4 years with frail conditions in Italy. Front Public Health. 2023 Jul 13;11:1212652.	
**140**	Ajose A, Akinde C, Ilo A, Durojaiye T, Shittu Y, Kadiri T, et al. Nigerian parents and caregivers knowledge, attitude and willingness to vaccinate their children against COVID-19. Front Public Health. 2023 Oct 2;11:1047285.	

**141**	Ng DL, Gan GG, Chai CS, Anuar NAB, Sindeh W, Chua WJ, et al. The willingness of parents to vaccinate their children younger than 12 years against COVID-19: a cross-sectional study in Malaysia. BMC Public Health. 2022 Jun 29;22(1):1265.	
**142**	Al-Iede M, Foudeh J, Al-Shweiki O, Alshrouf MA, Al-Abdallat T, Aleidi SM,et al. Parents' Willingness to Vaccinate Their Children Against COVID-19: A Cross-Sectional Survey From Jordan. Asia Pac J Public Health. 2022 Sep;34(6-7):698-701.	
**143**	Lam CN, Nicholas W, De La Torre A, Chan Y, Unger JB, Sood N, Hu H. Factors associated with parents' willingness to vaccinate their children against COVID-19: The LA pandemic surveillance cohort study. AIMS Public Health. 2022 May 25;9(3):482-489.	
**144**	Zheng M, Zhong W, Chen X, Wang N, Liu Y, Zhang Q, et al. Factors influencing parents' willingness to vaccinate their preschool children against COVID-19: Results from the mixed-method study in China. Hum Vaccin Immunother. 2022 Nov 30;18(6):2090776.	
**145**	Wong LP, Lee HY, Alias H, AbuBakar S. Malaysian Parents' Willingness to Vaccinate Their Children against COVID-19 Infection and Their Perception of mRNA COVID-19 Vaccines. Vaccines (Basel). 2022 Oct 25;10(11):1790.	
**146**	Swed S, Alibrahim H, Bohsas H, Shoib S, Hasan MM, Motawea KR, et al. Parents' acceptance to vaccinate children against COVID-19: A Syrian online survey. Front Public Health. 2022 Oct 13;10:955362.	
**147**	Santi T, Hegar B, Munasir Z, Prayitno A, Werdhani RA, Bandar INS, et al. Factors associated with parental intention to vaccinate their preschool children against COVID-19: a cross-sectional survey in urban area of Jakarta, Indonesia. Clin Exp Vaccine Res. 2023 Jul;12(3):240-248.	
**148**	Di Giuseppe G, Pelullo CP, Volgare AS, Napolitano F, Pavia M. Parents' Willingness to Vaccinate Their Children With COVID-19 Vaccine: Results of a Survey in Italy. J Adolesc Health. 2022 Apr;70(4):550-558.	
**149**	Lu L, Gu W, Xie H, Wang X, Cao L, Shan M, et al. Parental Attitudes Towards Vaccination Against COVID-19 in China During Pandemic. Infect Drug Resist. 2022 Aug 15;15:4541-4546.	
**150**	Wen LM, Xu H, Rissel C, Kerr E, Buchanan L, Taki S, et al. Demographic Predictors of Mothers' Willingness to Vaccinate Young Children Against COVID-19, Get Tested and Isolate: A Cross-Sectional Survey Before and During the Greater Sydney Lockdown 2021, Australia. Front Public Health. 2022 May 27;10:904495.	
**151**	Alsulaiman JW, Mazin M, Al-Shatanawi TN, Kheirallah KA, Allouh MZ. Parental Willingness to Vaccinate Their Children Against SARS-CoV-2 in Jordan: An Explanatory Cross-Sectional Study. Risk Manag Healthc Policy. 2022 May 10;15:955-967.	
**152**	Goldman RD, McGregor S, Marneni SR, Katsuta T, Griffiths MA, Hall JE, et al. Willingness to Vaccinate Children against Influenza after the Coronavirus Disease 2019 Pandemic. J Pediatr. 2021 Jan;228:87-93.e2.	
**153**	Goldman RD, Bone JN, Gelernter R, Krupik D, Ali S, Mater A, et al. National COVID-19 vaccine program progress and parents' willingness to vaccinate their children. Hum Vaccin Immunother. 2021 Dec 2;17(12):4889-4895.	
**154**	Postiglione M, Miraglia Del Giudice G, Della Polla G, Angelillo IF. Analysis of the COVID-19 vaccine willingness and hesitancy among parents of healthy children aged 6 months-4 years: a cross-sectional survey in Italy. Front Public Health. 2023 Oct 24;11:1241514.	
**155**	Krakowczyk JB, Bäuerle A, Pape L, Kaup T, Nulle L, Teufel M, et al. COVID-19 Vaccine for Children: Vaccination Willingness of Parents and Its Associated Factors-A Network Analysis. Vaccines (Basel). 2022 Jul 20;10(7):1155.	
**156**	Esposito S, Rosafio C, Partesotti S, Fiore M, Antodaro F, Bergomi A, et al. Knowledge on Parental Hesitancy toward COVID-19 Vaccination of Children 5-11 Years Old. Vaccines (Basel). 2023 Mar 3;11(3):587.	
**157**	Zhou Y, Li GX, Zhao TS, Du J, Zhang WX, Xie MZ, et al. Parents' willingness to vaccinate themselves and their children with the booster vaccine against SARS-CoV-2: A cross-sectional study in Puyang city, China. J Med Virol. 2023 Jan;95(1):e28256.	
**158**	Fernandes Nehab M, Gonçalves Camacho K, Teixeira Reis A, Junqueira-Marinho MF, Marques Abramov D, Almeida de Azevedo ZM, et al. Willingness of Brazilian caregivers in having their children and adolescents vaccinated against Covid-19. Vaccine. 2023 Jan 16;41(3):735-743.	
**159**	Yoda T, Katsuyama H. Parents' hesitation about getting their children vaccinated against COVID-19 in Japan. Hum Vaccin Immunother. 2021 Dec 2;17(12):4993-4998.	
**160**	Goldman RD, Bone JN, Gelernter R, Krupik D, Klein EJ, Griffiths MA, et al. Willingness to Accept Expedited COVID-19 Vaccine Research for Children Aged <12 Years After Adult Vaccine Approval. Clin Ther. 2022 Jan;44(1):e1-e10.	

**161**	Shmueli L. Has the COVID-19 Pandemic Changed Parental Attitudes and Beliefs Regarding Vaccinating Their Children against the Flu? Vaccines (Basel). 2023 Sep 24;11(10):1519.	
**162**	Carlson SJ, Attwell K, Roberts L, Hughes C, Blyth CC. West Australian parents' views on vaccinating their children against COVID-19: a qualitative study. BMC Public Health. 2023 Sep 11;23(1):1764.	
**163**	Wan X, Huang H, Shang J, Xie Z, Jia R, Lu G, et al. Willingness and influential factors of parents of 3-6-year-old children to vaccinate their children with the COVID-19 vaccine in China. Hum Vaccin Immunother. 2021 Nov 2;17(11):3969-3974.	
**164**	Choi SH, Jo YH, Jo KJ, Park SE. Pediatric and Parents' Attitudes Towards COVID-19 Vaccines and Intention to Vaccinate for Children. J Korean Med Sci. 2021 Aug 9;36(31):e227.	
**165**	Miraglia Del Giudice G, Napoli A, Corea F, Folcarelli L, Angelillo IF. Evaluating COVID-19 Vaccine Willingness and Hesitancy among Parents of Children Aged 5-11 Years with Chronic Conditions in Italy. Vaccines (Basel). 2022 Mar 4;10(3):396.	
**166**	Iannello P, Colautti L, Magenes S, Antonietti A, Cancer A. Black-and-white thinking and conspiracy beliefs prevent parents from vaccinating their children against COVID-19. Appl Cogn Psychol. 2022 Sep 21:10.1002/acp.3999.	
**167**	Huang LL, Tung TH, Jiang YH, Hu WW, Yang YP. Determinants of the willingness of medical staff to vaccinate their children with a booster dose of the COVID-19 vaccine in Taizhou, China. Hum Vaccin Immunother. 2022 Nov 30;18(6):2139098.	
**168**	AlHajri B, Alenezi D, Alfouzan H, Altamimi S, Alzalzalah S, Almansouri W, et al. Willingness of parents to vaccinate their children against influenza and the novel coronavirus disease-2019. J Pediatr. 2021 Apr;231:298-299.	
**169**	Darisi RD, Buckland AJ, Morales M, Ingram M, Harris E, Holzberg JR. Vaccine hesitancy and the willingness to recommend the COVID-19 vaccine to children in a rural country on the United States-Mexico border. Front Public Health. 2023 May 3;11:1127745.	
**170**	Ali-Saleh O, Khatib M, Hadid S, Dahamsheh K, Basis F. Factors Related to the Compliance of Arab Parents in Israel to the Vaccination of Children and Adolescents against COVID-19. Vaccines (Basel). 2023 Sep 28;11(10):1540.	
**171**	Ali-Saleh O, Bord S, Basis F. Factors Associated with Decisions of Arab Minority Parents in Israel to Vaccinate Their Children against COVID-19. Vaccines (Basel). 2022 May 29;10(6):870.	
**172**	Ruiz JB, Bell RA. Parental COVID-19 Vaccine Hesitancy in the United States. Public Health Rep. 2022 Nov-Dec;137(6):1162-1169.	
**173**	Hayat K, Farooq Umer M, Mujtaba H, Babar Kawish A, Azam Tahir M, Ullah Khan F, et al. Evaluation of Willingness to Accept COVID-19 Vaccine and Willingness to Pay among Pakistani Parents for Their Children Aged 5 to 11 Years: Findings and Implications. Am J Trop Med Hyg. 2023 May 15;109(1):69-75.	
**174**	Bourguiba A, AbuHijleh S, Nached Y, Waleed D, Farghaly S, AlOlama F. Assessing Parents' Knowledge, Attitudes, and Practices Toward Vaccinating Children (Five to 15 Years Old) Against COVID-19 in the United Arab Emirates. Cureus. 2022 Dec 17;14(12):e32625.	
**175**	Goldman RD, Marneni SR, Seiler M, Brown JC, Klein EJ, Cotanda CP, et al. Caregivers' Willingness to Accept Expedited Vaccine Research During the COVID-19 Pandemic: A Cross-sectional Survey. Clin Ther. 2020 Nov;42(11):2124-2133.	
**176**	Euser S, Kroese FM, Derks M, de Bruin M. Understanding COVID-19 vaccination willingness among youth: A survey study in the Netherlands. Vaccine. 2022 Feb 7;40(6):833-836.	
**177**	Bagateli LE, Saeki EY, Fadda M, Agostoni C, Marchisio P, Milani GP. COVID-19 Vaccine Hesitancy among Parents of Children and Adolescents Living in Brazil. Vaccines (Basel). 2021 Sep 30;9(10):1115.	
**178**	McKinnon B, Quach C, Dubé È, Tuong Nguyen C, Zinszer K. Social inequalities in COVID-19 vaccine acceptance and uptake for children and adolescents in Montreal, Canada. Vaccine. 2021 Dec 3;39(49):7140-7145.	
**179**	Wang Q, Xiu S, Zhao S, Wang J, Han Y, Dong S, et al. Vaccine Hesitancy: COVID-19 and Influenza Vaccine Willingness among Parents in Wuxi, China-A Cross-Sectional Study. Vaccines (Basel). 2021 Apr 1;9(4):342.	
**180**	Kocamaz EB, Kocamaz H. Awareness of Covid-19 and attitudes toward vaccination in parents of children between 0 and 18 years: A cross-sectional study. J Pediatr Nurs. 2022 Jul-Aug;65:75-81.	
**181**	Shaiba LA, Hadid A, Altirkawi K, Alnamnakani MA, Almutayliq AA, Alharbi AT, et al. SARS-CoV-2 Positivity in Early Infancy: A National Cohort From Saudi Arabia. Front Pediatr. 2022 Mar 28;10:849659.	

**182**	Özer M, Başkaya N, Bostancı İ. Attitudes towards influenza and pneumococcal vaccines in parents of asthmatic children during the COVID-19 pandemic. Pediatr Pulmonol. 2022 Apr;57(4):871-877.	
**183**	Goldman RD, Seiler M, Olson PG, Hart RJ, Bone JN, Baumer-Mouradian SH, et al. Factors associated with unvaccinated caregivers who plan to vaccinate their children. Prev Med. 2022 Sep;162:107121.	
**184**	Ma Y, Liu N, Zhong G, Wang D, Cao L, Bai S, et al. Parent Acceptance toward Inactivated COVID-19 Vaccination in Children with Acute Lymphoblastic Leukemia: The Power of Oncologist and Alliance. Vaccines (Basel). 2022 Nov 25;10(12):2016.	
**185**	Sarkar P, Chandrasekaran V, Gunasekaran D, Chinnakali P. COVID-19 vaccine hesitancy among health care worker-parents (HCWP) in Puducherry, India and its implications on their children: A cross sectional descriptive study. Vaccine. 2022 Sep 22;40(40):5821-5827.	
**186**	El-Shitany NA, Harakeh S, Badr-Eldin SM, Bagher AM, Eid B, Almukadi H, et al. Minor to Moderate Side Effects of Pfizer-BioNTech COVID-19 Vaccine Among Saudi Residents: A Retrospective Cross-Sectional Study. Int J Gen Med. 2021 Apr 19;14:1389-1401.	
**187**	Meraya AM, Salami RM, Alqahtani SS, Madkhali OA, Hijri AM, Qassadi FA, et al. AM. COVID-19 Vaccines and Restrictions: Concerns and Opinions among Individuals in Saudi Arabia. Healthcare (Basel). 2022 Apr 28;10(5):816.	
**188**	Della Polla G, Miraglia Del Giudice G, Postiglione M, Angelillo IF. Parents' Uptake and Willingness towards Recommended Vaccinations for Their Children with Underlying Chronic Medical Conditions in Italy. Vaccines (Basel). 2023 Aug 27;11(9):1423.	
**189**	Hart RJ, Baumer-Mouradian S, Bone JN, Olson P, Schroter S, Weigert RM, et al. Factors associated with US caregivers' uptake of pediatric COVID-19 vaccine by race and ethnicity. Vaccine. 2023 Apr 6;41(15):2546-2552.	
**190**	Bord S, Satran C, Schor A. The Mediating Role of the Perceived COVID-19 Vaccine Benefits: Examining Israeli Parents' Perceptions Regarding Their Adolescents' Vaccination. Vaccines (Basel). 2022 Jun 9;10(6):917.	
**191**	Kirui JC, Newberry DM, Harsh K. Strategies for Working With Parents With Vaccination Hesitancy. Neonatal Netw. 2023 Aug 1;42(5):254-263.	
**192**	Wang Q, Chen J, Jiang N, Zhang Y, Wang T, Cao H et al. Parents' intention to vaccinate their preschool children against COVID-19: Combining the health belief model and the theory of planned behavior. Hum Vaccin Immunother. 2023 Aug;19(2):2261171.	
**193**	Almojaibel AA, Ansari K, Alzahrani YA, Alessy SA, Farooqi FA, Alqurashi YD. Hesitancy towards the COVID-19 vaccine among health care practitioners in the Kingdom of Saudi Arabia: a cross-sectional study. F1000Res. 2023 Jun 12;11:24.	
**194**	Armitage R. GP confidence in counselling patients about COVID-19 vaccines: A cross-sectional survey. Public Health Pract (Oxf). 2021 Nov;2:100113.	
**195**	Ortiz-Martínez Y, López-López MÁ, Ruiz-González CE, Turbay-Caballero V, Sacoto DH, Caldera-Caballero M, et al. Willingness to receive COVID-19 vaccination in people living with HIV/AIDS from Latin America. Int J STD AIDS. 2022 Jun;33(7):652-659.	
**196**	Biharie A, Keuning MW, Wolthers KC, Pajkrt D. Comorbidities, clinical characteristics and outcomes of COVID-19 in pediatric patients in a tertiary medical center in the Netherlands. World J Pediatr. 2022 Aug;18(8):558-563.	
**197**	Özdemir Ö, Dikici Ü. COVID-19 vaccination rate and side effects in CVID patients over the age of 12. Hum Vaccin Immunother. 2022 Nov 30;18(5):2068337.	
**198**	Hsiao SH, Huang SJ, Huang CY. Vaccination Strategies at a COVID-19 Mass Vaccination Site. Int J Health Policy Manag. 2022 Sep 1;11(9):1981-1982.	
**199**	Huddleston HG, Jaswa EG, Lindquist KJ, Kaing A, Morris JR, Hariton E, et al. COVID-19 vaccination patterns and attitudes among American pregnant individuals. Am J Obstet Gynecol MFM. 2022 Jan;4(1):100507.	
**200**	Barry M, Temsah MH, Alhuzaimi A, Alamro N, Al-Eyadhy A, Aljamaan F, et al. COVID-19 vaccine confidence and hesitancy among health care workers: A cross-sectional survey from a MERS-CoV experienced nation. PLoS One. 2021 Nov 29;16(11):e0244415.	
**201**	Moeller KE, Meeks M, Reynoldson J, Douglass M. Implementation and Outcomes of COVID-19 Vaccinations at a Child and Adolescent Psychiatric Hospital. J Am Acad Child Adolesc Psychiatry. 2021 Nov;60(11):1332-1334.	
**202**	Kohler RE, Wagner RB, Careaga K, Vega J, Btoush R, Greene K, et al. Parents' Intentions, Concerns and Information Needs about COVID-19 Vaccination in New Jersey: A Qualitative Analysis. Vaccines (Basel). 2023 Jun 13;11(6):1096.	

**203**	Cao M, Zhao J, Huang C, Wang X, Ye L, Han X, et al. Assessing vaccine hesitancy using the WHO scale for caregivers of children under 3 years old in China. Front Public Health. 2023 Apr 12;11:1090609.	
**204**	Rungkitwattanakul D, Yabusaki A, Singh D, Lawson P, Nwaogwugwu U, Iheagwara OS, et al. COVID-19 vaccine hesitancy among African American hemodialysis patients: A single-center experience. Hemodial Int. 2021 Mar 11;25(3):410–2.	
**205**	Fadl N, Al Awaidy ST, Elshabrawy A, Makhlouf MSAH, Ibrahim SA, Abdel-Rahman S, et al. Determinants of parental seasonal influenza vaccine hesitancy in the Eastern Mediterranean region: A cross-sectional study. Front Public Health. 2023 Mar 28;11:1132798.	
**206**	Ateş BÖ, Özyavuz G, Cöngöloğlu MA. COVID-19 vaccine hesitancy of adolescents with psychiatric disorders and their parents: data from a child psychiatry outpatient clinic. Turk J Pediatr. 2023 April 25;65(2):205-217.	
**207**	Stephens AB, Hofstetter AM, Stockwell MS. Influenza Vaccine Hesitancy: Scope, Influencing Factors, and Strategic Interventions. Pediatr Clin North Am. 2023 Apr;70(2):227-241.	
**208**	Šašić M, Bodulić K, Hojsak I, Mašić M, Trivić I, Markić J, et al. Parents' attitudes toward childhood COVID-19 immunization in Croatia: a multicenter cross-sectional study. Croat Med J. 2023 Feb 28;64(1):52-60.	
**209**	Puleh SS, Kigongo E, Opio IO, Akech SI, Opollo MS, Achan E, et al. Parents' Readiness to Vaccinate Their Children Aged 5 to 17 Years Against Covid-19 and Its Associated Factors in Lira District, Uganda. Pediatric Health Med Ther. 2023 Apr 20;14:131-139.	
**210**	Obasanya M, Igenoza O, Gupta S, McElroy K, Brannon GE, Brown K. Racial and Ethnic Differences in Maternal and Child COVID-19 Vaccination Intent Among Pregnant and Postpartum Women in the USA (April-June 2020): an Application of Health Belief Model. J Racial Ethn Health Disparities. 2023 Oct;10(5):2540-2551.	
**211**	Shkalim Zemer V, Grossman Z, Cohen HA, Hoshen M, Gerstein M, Richenberg Y, et al. Variables Associated With COVID-19 Vaccination Among Israeli Adolescents and the Need for Targeted Interventions. Pediatr Infect Dis J. 2022 Nov 1;41(11):927-932.	
**212**	Samudyatha UC, Balaji B, Singh M, Gowda M. Caregivers' Preferences of COVID-19 Vaccination for Children: A Cross-sectional Study From Rural South India. Medeni Med J. 2022 Sep 21;37(3):248-254.	
**213**	Napoli A, Miraglia Del Giudice G, Corea F, Folcarelli L, Angelillo IF. Parents' reasons to vaccinate their children aged 5-11 years against COVID-19 in Italy. Front Med (Lausanne). 2022 Aug 2;9:949693.	
**214**	Hou Z, Song K, Wang Q, Zang S, Tu S, Chantler T, et al. Childhood COVID-19 vaccine acceptance and preference from caregivers and healthcare workers in China: A survey experiment. Prev Med. 2022 Aug;161:107138.	
**215**	Shen X, Wu X, Deng Z, Liu X, Zhu Y, Huang Y, et al. Analysis on vaccine hesitation and its associated factors among parents of preschool children in Songgang Street, Shenzhen. Sci Rep. 2022 Jun 8;12(1):9467.	
**216**	Wang Q, Xiu S, Yang L, Han Y, Cui T, Shi N, et al. Changes in Parental Attitudes Toward COVID-19 Vaccination and Routine Childhood Vaccination During the COVID-19 Pandemic: Repeated Cross-sectional Survey Study. JMIR Public Health Surveill. 2022 May 13;8(5):e33235.	
**217**	Mohan R, Pandey V, Kumar A, Gangadevi P, Goel AD, Joseph J, et al. Acceptance and Attitude of Parents Regarding COVID-19 Vaccine for Children: A Cross-Sectional Study. Cureus. 2022 Apr 27;14(4):e24518.	
**218**	Faye SLB, Krumkamp R, Doumbia S, Tounkara M, Strauss R, Ouedraogo HG, et al. Factors influencing hesitancy towards adult and child COVID-19 vaccines in rural and urban West Africa: a cross-sectional study. BMJ Open. 2022 Apr 13;12(4):e059138.	
**219**	Wagner A, Liberatore F, Schmelzer S, Dratva J. Confident and altruistic - parents' motives to vaccinate their children against COVID-19: a cross-sectional online survey in a Swiss vaccination centre. Swiss Med Wkly. 2022 Mar 18;152:w30156.	
**220**	Koh SWC, Tan HM, Lee WH, Mathews J, Young D. COVID-19 Vaccine Booster Hesitancy among Healthcare Workers: A Retrospective Observational Study in Singapore. Vaccines (Basel). 2022 Mar 17;10(3):464.	
**221**	Sabahelzain MM, Moukhyer M, van den Borne B, Bosma H. Vaccine Hesitancy among Parents and Its Association with the Uptake of Measles Vaccine in Urban Settings in Khartoum State, Sudan. Vaccines (Basel). 2022 Jan 28;10(2):205.	
**222**	Sutan R, Batarfi SA, Ismail H, Bin-Ghouth AS. Vaccine hesitancy from parents and healthcare providers perspectives in Hadhramout Governorate, Yemen: a mixed-method study protocol. BMJ Open. 2022 Feb 17;12(2):e055841.	

**223**	Sabahelzain MM, Moukhyer M, Bosma H, van den Borne B. Determinants of Measles Vaccine Hesitancy among Sudanese Parents in Khartoum State, Sudan: A Cross-Sectional Study. Vaccines (Basel). 2021 Dec 22;10(1):6.	
**224**	Zhang H, Zheng P, Zhang J, Qiu Q, Huang B, Feng H, et al. Vaccine hesitancy among parents and its influencing factors: a cross-sectional study in Guangzhou, China. Hum Vaccin Immunother. 2021 Dec 2;17(12):5153-5161.	
**225**	Griva K, Tan KYK, Chan FHF, Periakaruppan R, Ong BWL, Soh ASE, et al. Evaluating Rates and Determinants of COVID-19 Vaccine Hesitancy for Adults and Children in the Singapore Population: Strengthening Our Community's Resilience against Threats from Emerging Infections (SOCRATEs) Cohort. Vaccines (Basel). 2021 Nov 30;9(12):1415.	
**226**	Patwary MM, Bardhan M, Disha AS, Hasan M, Haque MZ, Sultana R, et al. Determinants of COVID-19 Vaccine Acceptance among the Adult Population of Bangladesh Using the Health Belief Model and the Theory of Planned Behavior Model. Vaccines (Basel). 2021 Nov 25;9(12):1393.	
**227**	Noushad M, Nassani MZ, Al-Awar MS, Al-Saqqaf IS, Mohammed SOA, Samran A, et al. COVID-19 Vaccine Hesitancy Associated With Vaccine Inequity Among Healthcare Workers in a Low-Income Fragile Nation. Front Public Health. 2022 Jul 11;10:914943.	
**228**	Zhang MX, Lin XQ, Chen Y, Tung TH, Zhu JS. Determinants of parental hesitancy to vaccinate their children against COVID-19 in China. Expert Rev Vaccines. 2021 Oct;20(10):1339-1349.	
**229**	Gentile A, Pacchiotti AC, Giglio N, Nolte MF, Talamona N, Rogers V, et al. Vaccine hesitancy in Argentina: Validation of WHO scale for parents. Vaccine. 2021 Jul 30;39(33):4611-4619.	
**230**	Reno C, Maietti E, Fantini MP, Savoia E, Manzoli L, Montalti M, et al. Enhancing COVID-19 Vaccines Acceptance: Results from a Survey on Vaccine Hesitancy in Northern Italy. Vaccines (Basel). 2021 Apr 13;9(4):378.	
**231**	Ryan GW, Askelson NM, Woodworth KR, Lindley MC, Gedlinske A, Parker AM, et al. Unvaccinated Adolescents' COVID-19 Vaccine Intentions: Implications for Public Health Messaging. J Adolesc Health. 2023 Oct;73(4):679-685.	
**232**	Yasmin F, Kumari K, Saleem K, Lareeb I, Shaikh A, Ashfaq R, et al. Caregiver's perceptions of COVID-19 vaccination, and intention to vaccinate their children against the disease: a questionnaire based qualitative study. Ann Med Surg (Lond). 2023 Aug 9;85(10):4757-4763.	
**233**	Akhtar N, Dash GC, Kumawat A, Parai D, Choudhary HR, Mohanta AR, et al. COVID-19 vaccine hesitancy among school children aged 12-14 years: A cross-sectional study from Bhubaneswar, Odisha, India. J Infect Dev Ctries. 2023 May 31;17(5):583-587.	
**234**	Alshehry AS, Cruz JP, Alquwez N, Alsharari AF, Tork HMM, Almazan JU, et al. Predictors of nursing students' intention to receive COVID-19 vaccination: A multi-university study in Saudi Arabia. J Adv Nurs. 2022 Feb;78(2):446-457.	
**235**	Deng JS, Chen JY, Lin XQ, Huang CL, Tung TH, Zhu JS. Parental hesitancy against COVID-19 vaccination for children and associated factors in Taiwan. BMC Public Health. 2023 Mar 27;23(1):571.	
**236**	Shmueli L. Parents' intention to vaccinate their 5- to 11-year-old children with the COVID-19 vaccine: rates, predictors and the role of incentives. BMC Public Health. 2023 Feb 14;23(1):328.	
**237**	Xia Y, Li Q, Jiao W, Lan Y. Dynamic mechanism of eliminating COVID-19 vaccine hesitancy through web search. Front Public Health. 2023 Jan 30;11:1018378.	
**238**	Yuan S, Rui J, Peng X. Trust in scientists on COVID-19 vaccine hesitancy and vaccine intention in China and the US. Int J Disaster Risk Reduct. 2023 Feb 15;86:103539.	
**239**	Cho HK, Lee H, Choe YJ, Kim S, Seo S, Moon J, et al. Parental concerns about COVID-19 vaccine safety and hesitancy in Korea: implications for vaccine communication. Epidemiol Health. 2022;45:e2023004.	
**240**	Zhang K, Liang X, Tam KLW, Kawuki J, Chan PS, Chen S, et al. Changes in COVID-19 Vaccine Acceptability among Parents with Children Aged 6-35 Months in China-Repeated Cross-Sectional Surveys in 2020 and 2021. Vaccines (Basel). 2023 Jan 12;11(1):170.	
**241**	Vasudevan L, Stinnett SS, Hart L, Gomez Altamirano P, Gonzalez A, Weaver K, et al. Pregnant individuals' information needs and intention to vaccinate their children with routine and COVID-19 vaccines: Findings from a cross-sectional survey. Int J Gynaecol Obstet. 2023 Jul;162(1):78-87.	
**242**	Baumer-Mouradian SH, Hart RJ, Visotcky A, Fraser R, Prasad S, Levas M, et al. Understanding Influenza and SARS-CoV-2 Vaccine Hesitancy in Racial and Ethnic Minority Caregivers. Vaccines (Basel). 2022 Nov 20;10(11):1968.	

**243**	Askarian M, Semenov A, Llopis F, Rubulotta F, Dragovac G, Pshenichnaya et al. The COVID-19 vaccination acceptance/hesitancy rate and its determinants among healthcare workers of 91 Countries: A multicenter cross-sectional study. EXCLI J. 2022 Jan 6;21:93-103.	
**244**	Martinez EZ, Zucoloto ML, Ramos VP, Dutra CDC, de Jesus GJ, Esteves AVF, et al. Brazilian Adults' Attitudes and Practices Regarding the Mandatory COVID-19 Vaccination and Their Hesitancy towards Childhood Vaccination. Vaccines (Basel). 2022 Nov 1;10(11):1853.	
**245**	Sayed AA. Assessing the Impact of Use and Trust in Different Sources of Information on COVID-19 Vaccination Uptake in Saudi Arabia (SA) Using the COVID-19 Vaccine Hesitancy and Resistance in SA (CoV-HERSA) Tool. Trop Med Infect Dis. 2022 Nov 14;7(11):375.	
**246**	Byrne A, Thompson LA, Filipp SL, Ryan K. COVID-19 vaccine perceptions and hesitancy amongst parents of school-aged children during the pediatric vaccine rollout. Vaccine. 2022 Nov 2;40(46):6680-6687.	
**247**	Debela MS, Garrett APN, Charania NA. Vaccine hesitancy and its determinants among refugee parents resettled in Aotearoa New Zealand. Hum Vaccin Immunother. 2022 Nov 30;18(6):2131336.	
**248**	Panchalingam T, Shi Y. Parental refusal and hesitancy of vaccinating children against COVID-19: Findings from a nationally representative sample of parents in the U.S. Prev Med. 2022 Nov;164:107288.	
**249**	Low JM, Soo CWT, Phuong TA, Zhong Y, Lee LY. Predicting vaccine hesitancy among parents towards COVID-19 vaccination for their children in Singapore. Front Pediatr. 2022 Oct 10;10:994675.	
**250**	Lin XQ, Li AL, Zhang MX, Lv L, Chen Y, Chen HD, et al. Willingness of Older Adults with Chronic Diseases to Receive a Booster Dose of Inactivated Coronavirus Disease 2019 Vaccine: A Cross-Sectional Study in Taizhou, China. Vaccines (Basel). 2022 Oct 6;10(10):1665.	
**251**	Ulaszewska K, Jodczyk AM, Długołęcki P, Emerla S, Stańska W, Kasiak PS, et al. Factors Associated with Willingness to Receive a COVID-19 Vaccine in Adult Polish Population-A Cross-Sectional Survey. Vaccines (Basel). 2022 Oct 14;10(10):1715.	
**252**	Goulding M, Ryan GW, Minkah P, Borg A, Gonzalez M, Medina N, et al. Parental perceptions of the COVID-19 vaccine for 5- to 11-year-old children: Focus group findings from Worcester Massachusetts. Hum Vaccin Immunother. 2022 Nov 30;18(6):2120721.	
**253**	AlKetbi LMB, Al Hosani F, Al Memari S, Al Mazrouei S, Al Shehhi B, AlShamsi N, et al. Parents' views on the acceptability of a COVID-19 vaccine for their children: A cross-sectional study in Abu Dhabi-United Arab Emirates. Vaccine. 2022 Sep 9;40(38):5562-5568.	
**254**	Kyei-Arthur F, Kyei-Gyamfi S, Agyekum MW, Afrifa-Anane GF, Amoh BA. Parents' and guardians' acceptability of COVID-19 vaccination for children in Ghana: An online survey. PLoS One. 2022 Aug 29;17(8):e0272801.	
**255**	Benites-Zapata VA, Herrera-Añazco P, Benites-Meza JK, Bonilla-Aguilar K, Urrunaga-Pastor D, Bendezu-Quispe G, et al. Prevalence of parents' non-intention to vaccinate their children and adolescents against COVID-19: A comparative analysis in Colombia and Peru. Vaccine X. 2022 Jul 27;12:100198.	
**256**	Fisher CB, Bragard E, Jaber R, Gray A. COVID-19 Vaccine Hesitancy among Parents of Children under Five Years in the United States. Vaccines (Basel). 2022 Aug 14;10(8):1313.	
**257**	Ryalat S, Alduraidi H, Al-Ryalat SA, Alzu'bi M, Alzyoud M, Odeh N, et al. Attitudes towards COVID-19 Booster Vaccines, Vaccine Preferences, Child Immunization, and Recent Issues in Vaccination among University Students in Jordan. Vaccines (Basel). 2022 Aug 4;10(8):1258.	
**258**	Siewchaisakul P, Sarakarn P, Nanthanangkul S, Longkul J, Boonchieng W, Wungrath J. Role of literacy, fear and hesitancy on acceptance of COVID-19 vaccine among village health volunteers in Thailand. PLoS One. 2022 Jun 24;17(6):e0270023.	
**259**	Hammershaimb EA, Cole LD, Liang Y, Hendrich MA, Das D, Petrin R, et al. COVID-19 Vaccine Acceptance Among US Parents: A Nationally Representative Survey. J Pediatric Infect Dis Soc. 2022 Aug 30;11(8):361-370.	
**260**	Lecce M, Milani GP, Agostoni C, D'Auria E, Banderali G, Biganzoli G, et al. Caregivers' Intention to Vaccinate Their Children Under 12 Years of Age Against COVID-19: A Cross-Sectional Multi-Center Study in Milan, Italy. Front Pediatr. 2022 May 30;10:834363.	

**261**	Simkhada P, Tamang P, Timilsina L, Simkhada B, Bissell P, van Teijlingen E, et al. Factors Influencing COVID-19 Vaccine Uptake among Nepali People in the UK: A Qualitative Study. Vaccines (Basel). 2022 May 14;10(5):780.	
**262**	Lau EYH, Li JB, King Chung Chan D. Intention to vaccinate young children against COVID-19: A large-scale survey of Hong Kong parents. Hum Vaccin Immunother. 2022 Nov 30;18(5):2065838.	
**263**	Letterie MC, Patrick SW, Halvorson AE, Dupont WD, Carroll KN, Zickafoose JS, et al. Factors Associated With Parental COVID-19 Vaccination Acceptance. Clin Pediatr (Phila). 2022 Jun;61(5-6):393-401.	
**264**	Ji M, Huang Z, Ren J, Wagner AL. Vaccine hesitancy and receipt of mandatory and optional pediatric vaccines in Shanghai, China. Hum Vaccin Immunother. 2022 Nov 30;18(5):2043025.	
**265**	McElfish PA, Willis DE, Shah SK, Reece S, Andersen JA, Schootman M, et al. Parents' and Guardians' Intentions to Vaccinate Children against COVID-19. Vaccines (Basel). 2022 Feb 25;10(3):361.	
**266**	Ellithorpe ME, Aladé F, Adams RB, Nowak GJ. Looking ahead: Caregivers' COVID-19 vaccination intention for children 5 years old and younger using the health belief model. Vaccine. 2022 Mar 1;40(10):1404-1412.	
**267**	Huang Y, Su X, Xiao W, Wang H, Si M, Wang W, et al. COVID-19 vaccine hesitancy among different population groups in China: a national multicenter online survey. BMC Infect Dis. 2022 Feb 14;22(1):153.	
**268**	Marzo RR, Sami W, Alam MZ, Acharya S, Jermsittiparsert K, Songwathana K, et al. Hesitancy in COVID-19 vaccine uptake and its associated factors among the general adult population: a cross-sectional study in six Southeast Asian countries. Trop Med Health. 2022 Jan 5;50(1):4.	
**269**	Bianco A, Della Polla G, Angelillo S, Pelullo CP, Licata F, Angelillo IF. Parental COVID-19 vaccine hesitancy: a cross-sectional survey in Italy. Expert Rev Vaccines. 2022 Apr;21(4):541-547.	
**270**	Galanis P, Vraka I, Siskou O, Konstantakopoulou O, Katsiroumpa A, Moisoglou I, et al. Predictors of Real-World Parents' Acceptance to Vaccinate Their Children Against the COVID-19. Acta Med Litu. 2022;29(1):33-43.	
**271**	Sinuraya RK, Kusuma ASW, Pardoel ZE, Postma MJ, Suwantika AA. Parents' Knowledge, Attitude, and Practice on Childhood Vaccination During the COVID-19 Pandemic in Indonesia. Patient Prefer Adherence. 2022 Jan 14;16:105-112.	
**272**	Vizcardo D, Salvador LF, Nole-Vara A, Dávila KP, Alvarez-Risco A, Yáñez JA, et al. Sociodemographic Predictors Associated with the Willingness to Get Vaccinated against COVID-19 in Peru: A Cross-Sectional Survey. Vaccines (Basel). 2021 Dec 30;10(1):48.	
**273**	Wang Y, Zhang X. Influence of Parental Psychological Flexibility on Pediatric COVID-19 Vaccine Hesitancy: Mediating Role of Self-Efficacy and Coping Style. Front Psychol. 2021 Dec 8;12:783401.	
**274**	Bari MS, Hossain MJ, Ahmmed F, Sarker MMR, Khandokar L, Chaithy AP, et al. Knowledge, Perception, and Willingness towards Immunization among Bangladeshi Population during COVID-19 Vaccine Rolling Period. Vaccines (Basel). 2021 Dec 7;9(12):1449.	
**275**	Hara M, Ishibashi M, Nakane A, Nakano T, Hirota Y. Differences in COVID-19 Vaccine Acceptance, Hesitancy, and Confidence between Healthcare Workers and the General Population in Japan. Vaccines (Basel). 2021 Nov 24;9(12):1389.	
**276**	Roberts CH, Brindle H, Rogers NT, Eggo RM, Enria L, Lees S. Vaccine Confidence and Hesitancy at the Start of COVID-19 Vaccine Deployment in the UK: An Embedded Mixed-Methods Study. Front Public Health. 2021 Nov 11;9:745630.	
**277**	Feng H, Zhu H, Zhang H, Cao L, Li L, Wang J, et al. Caregivers' intentions to COVID-19 vaccination for their children in China: a cross-sectional survey. Hum Vaccin Immunother. 2021 Dec 2;17(12):4799-4805.	
**278**	Babicki M, Pokorna-Kałwak D, Doniec Z, Mastalerz-Migas A. Attitudes of Parents with Regard to Vaccination of Children against COVID-19 in Poland. A Nationwide Online Survey. Vaccines (Basel). 2021 Oct 17;9(10):1192.	
**279**	Du M, Tao L, Liu J. The Association Between Risk Perception and COVID-19 Vaccine Hesitancy for Children Among Reproductive Women in China: An Online Survey. Front Med (Lausanne). 2021 Sep 8;8:741298.	
**280**	He K, Mack WJ, Neely M, Lewis L, Anand V. Parental Perspectives on Immunizations: Impact of the COVID-19 Pandemic on Childhood Vaccine Hesitancy. J Community Health. 2022 Feb;47(1):39-52.	
**281**	Fadda M, Suggs LS, Albanese E. Willingness to vaccinate against Covid-19: A qualitative study involving older adults from Southern Switzerland. Vaccine X. 2021 Aug;8:100108.	

**282**	Shen X, Dong H, Feng J, Jiang H, Dowling R, Lu Z, et al. Assessing the COVID-19 vaccine hesitancy in the Chinese adults using a generalized vaccine hesitancy survey instrument. Hum Vaccin Immunother. 2021 Nov 2;17(11):4005-4012.	
**283**	Fedele F, Aria M, Esposito V, Micillo M, Cecere G, Spano M, et al. COVID-19 vaccine hesitancy: a survey in a population highly compliant to common vaccinations. Hum Vaccin Immunother. 2021 Oct 3;17(10):3348-3354.	
**284**	Yılmaz M, Sahin MK. Parents' willingness and attitudes concerning the COVID-19 vaccine: A cross-sectional study. Int J Clin Pract. 2021 Sep;75(9):e14364.	
**285**	Brandstetter S, Böhmer MM, Pawellek M, Seelbach-Göbel B, Melter M, Kabesch M, et al. Parents' intention to get vaccinated and to have their child vaccinated against COVID-19: cross-sectional analyses using data from the KUNO-Kids health study. Eur J Pediatr. 2021 Nov;180(11):3405-3410.	
**286**	Kukreti S, Lu MY, Lin YH, Strong C, Lin CY, Ko NY, et al. Willingness of Taiwan's Healthcare Workers and Outpatients to Vaccinate against COVID-19 during a Period without Community Outbreaks. Vaccines (Basel). 2021 Mar 12;9(3):246.	
**287**	Sallam M, Dababseh D, Eid H, Al-Mahzoum K, Al-Haidar A, Taim D, et al. High Rates of COVID-19 Vaccine Hesitancy and Its Association with Conspiracy Beliefs: A Study in Jordan and Kuwait among Other Arab Countries. Vaccines (Basel). 2021 Jan 12;9(1):42.	
**288**	Lazarus JV, Wyka K, Rauh L, Rabin K, Ratzan S, Gostin LO, et al. Hesitant or Not? The Association of Age, Gender, and Education with Potential Acceptance of a COVID-19 Vaccine: A Country-level Analysis. J Health Commun. 2020 Oct 2;25(10):799-807.	
**289**	Alsuwaidi AR, Elbarazi I, Al-Hamad S, Aldhaheri R, Sheek-Hussein M, Narchi H. Vaccine hesitancy and its determinants among Arab parents: a cross-sectional survey in the United Arab Emirates. Hum Vaccin Immunother. 2020 Dec 1;16(12):3163-3169.	
**290**	AlGethami HJ, Altamran MA, Khan MS, Zaman KMN, Alswaied N. Awareness and Knowledge Towards Pediatric and Adult COVID-19 Vaccination: A Cross Sectional Community-based Study in Saudi Arabia. Mater Sociomed. 2021 Dec;33(4):262-268.	
**291**	Momani A, Hamaideh SH, Masadeh AB, Alhalaiqa F, Bani Mostafa FN, Weld Ali HI, et al. The effect of COVID-19 vaccine tele-educational program on vaccine hesitancy and receiving the vaccine among women planning for pregnancy, pregnant or breast-feeding mothers. PLoS One. 2023 Mar 9;18(3):e0282627.	
**292**	Njororai F, Nyaranga KC, Cholo W, Amulla W, Ndetan H. Correlates of COVID-19 Vaccine Acceptance and Hesitancy in Rural Communities in Western Kenya. Vaccines (Basel). 2023 Sep 23;11(10):1516.	
**293**	Habbash AS, Siddiqui AF. Factors Affecting COVID-19 Vaccine Acceptance among Pregnant Women: A Cross Sectional Study from Abha City, Saudi Arabia. Vaccines (Basel). 2023 Sep 7;11(9):1463.	
**294**	Ghazy RM, Ibrahim SA, Taha SHN, Elshabrawy A, Elkhadry SW, Abdel-Rahman S, et al. Attitudes of parents towards influenza vaccine in the Eastern Mediterranean Region: A multilevel analysis. Vaccine. 2023 Aug 14;41(36):5253-5264.	
**295**	Hilton Z, Hoq M, Danchin M, Kaufman J. Reducing COVID-19 Vaccine Decisional Conflict in Parents of 5-11-Year-Old Children in Australia: A Single Arm Pre-Post Study. Vaccines (Basel). 2023 Jul 28;11(8):1296.	
**296**	Omar SM, Osman OS, Khalil R, Al-Wutayd O, Adam I. COVID-19 vaccine acceptance among pregnant women: a hospital-based cross-sectional study in Sudan. Front Public Health. 2023 Jul 17;11:1221788.	
**297**	Lin CY, Hsiao RC, Chen YM, Yen CF. A Parent Version of the Motors of COVID-19 Vaccination Acceptance Scale for Assessing Parents' Motivation to Have Their Children Vaccinated. Vaccines (Basel). 2023 Jul 3;11(7):1192.	
**298**	Zengeni M, Briggs NN. The Prevalence of COVID-19 Vaccine Hesitancy Among the Black Asian Ethnic Minority in New South Wales, Australia. Cureus. 2023 Jun 19;15(6):e40626.	
**299**	Miral MT, Turgut N, Güldür A, Güloğlu ZE, Mamuk R. COVID-19 fear, vaccination hesitancy, and vaccination status in pregnant and breastfeeding women in Turkey. Afr J Reprod Health. 2023 Jun;27(6):60-69.	
**300**	Rojop N, Calvimontes DM, Barrios E, Lamb MM, Paniagua-Avila A, Monzon J, et al. COVID-19 Attitudes and Vaccine Hesitancy among an Agricultural Community in Southwest Guatemala: A Cross-Sectional Survey. Vaccines (Basel). 2023 Jun 2;11(6):1059.	
**301**	Tekin Ç, Gökçe A, Boz G, Aslan M, Yiğit E. Reasons for parental hesitancy or refusal of childhood vaccination in Türkiye. East Mediterr Health J. 2023 May 31;29(5):343-353.	
**302**	Moon I, Han J, Kim K. Determinants of COVID-19 vaccine Hesitancy: 2020 California Health Interview Survey. Prev Med Rep. 2023 Jun;33:102200.	

**303**	Wigle J, Hodwitz K, Juando-Prats C, Allan K, Li X, Howard L, et al. Parents' perspectives on SARS-CoV-2 vaccinations for children: a qualitative analysis. CMAJ. 2023 Feb 21;195(7):E259-E266.	
**304**	Davidson CA, Jackson KT, Kennedy K, Stoyanovich E, Mantler T. Vaccine Hesitancy Among Canadian Mothers: Differences in Attitudes Towards a Pediatric COVID-19 Vaccine Among Women Who Experience Intimate Partner Violence. Matern Child Health J. 2023 Mar;27(3):566-574.	

Non-eligible Articles/ Records excluded because studies are not cross-sectional (reviews, systematic reviews) (n=35)

**Serial**	**Article**	**Remarks**
**1**	Iqbal MS, Khan SU, Qamer S, Vohra S. Vaccine Hesitancy of COVID-19 among Parents for Their Children in Middle Eastern Countries-A Systematic Review. Vaccines (Basel). 2023 Oct 1;11(10):1556.	Systematic review
**2**	Algabbani A, AlOmeir O, Algabbani F. Vaccine hesitancy in the Gulf Cooperation Council countries. East Mediterr Health J. 2023 May 31;29(5):402-411.	Systematic review
**3**	Rajeh MT, Farsi DJ, Farsi NJ, Mosli HH, Mosli MH. Are parents' willing to vaccinate their children against COVID-19? A qualitative study based on the Health Belief Model. Hum Vaccin Immunother. 2023 31;19(1):2177068. doi: 10.1080/21645515.2023.2177068. Epub 2023 Feb 8.	Qualitative study<br>Epub 2023 Feb 8.
**4**	Fajar JK, Sallam M, Soegiarto G, Sugiri YJ, Anshory M, Wulandari L, et al. Global Prevalence and Potential Influencing Factors of COVID-19 Vaccination Hesitancy: A Meta-Analysis. Vaccines (Basel). 2022 Aug 19;10(8):1356.	Meta-Analysis
**5**	Alimoradi Z, Lin CY, Pakpour AH. Worldwide Estimation of Parental Acceptance of COVID-19 Vaccine for Their Children: A Systematic Review and Meta-Analysis. Vaccines (Basel). 2023 Feb 24;11(3):533.	Systematic Review and Meta-Analysis
**6**	Aldossari HM. Exploring interrelationships of COVID-19 dimensions in Saudi Arabia: a systematic review. Bull Natl Res Cent. 2023;47(1):73.	Systematic review<br>Epub 2023 May 26.
**7**	Alabadi M, Aldawood Z. Parents' Knowledge, Attitude and Perceptions on Childhood Vaccination in Saudi Arabia: A Systematic Literature Review. Vaccines (Basel). 2020 Dec 10;8(4):750.	Systematic review
**8**	Wang Y, Zhang X. Key factors influencing paediatric COVID-19 vaccine hesitancy: a brief overview and Decision-making Trial and Evaluation Laboratory analysis. Public Health. 2023 May;218:97-105.	Systematic review
**9**	Norhayati MN, Che Yusof R, Azman YM. Systematic Review and Meta-Analysis of COVID-19 Vaccination Acceptance. Front Med (Lausanne). 2022 Jan 27;8:783982.	Systematic Review and Meta-Analysis
**10**	Renzi E, Baccolini V, Migliara G, Bellotta C, Ceparano M, Donia P, et al. Mapping the Prevalence of COVID-19 Vaccine Acceptance at the Global and Regional Level: A Systematic Review and Meta-Analysis. Vaccines (Basel). 2022 Sep 7;10(9):1488.	Systematic Review and Meta-Analysis
**11**	Salomoni MG, Di Valerio Z, Gabrielli E, Montalti M, Tedesco D, Guaraldi F, Gori D. Hesitant or Not Hesitant? A Systematic Review on Global COVID-19 Vaccine Acceptance in Different Populations. Vaccines (Basel). 2021 Aug 6;9(8):873.	Systematic review
**12**	Alam Z, Mohamed S, Nauman J, Al-Rifai RH, Ahmed LA, Elbarazi I. Hesitancy toward vaccination against COVID-19: A scoping review of prevalence and associated factors in the Arab world. Hum Vaccin Immunother. 2023 Aug 1;19(2):2245720.	Scoping review
**13**	Sheerah HA, Almuzaini Y, Khan A. Public Health Challenges in Saudi Arabia during the COVID-19 Pandemic: A Literature Review. Healthcare (Basel). 2023 Jun 15;11(12):1757.	Literature Review
**14**	Limbu YB, Gautam RK. The determinants of COVID-19 vaccination intention: a meta-review. Front Public Health. 2023 Jun 12;11:1162861.	Meta-Review
**15**	Khan YH, Rasheed M, Mallhi TH, Salman M, Alzarea AI, Alanazi AS, et al. Barriers and facilitators of childhood COVID-19 vaccination among parents: A systematic review. Front Pediatr. 2022 Nov 24;10:950406.	Systematic review
**16**	Obohwemu K, Christie-de Jong F, Ling J. Parental childhood vaccine hesitancy and predicting uptake of vaccinations: a systematic review. Prim Health Care Res Dev. 2022 Nov 4;23:e68.	Systematic review
**17**	Chen F, He Y, Shi Y. Parents' and Guardians' Willingness to Vaccinate Their Children against COVID-19: A Systematic Review and Meta-Analysis. Vaccines (Basel). 2022 Jan 24;10(2):179.	Systematic Review and Meta-Analysis

**18**	Zhang M, Zhang P, Liang Y, Du B, Li L, Yu Z, et al. A systematic review of current status and challenges of vaccinating children against SARS-CoV-2. J Infect Public Health. 2022 Nov;15(11):1212-1224.	Systematic review
**19**	Liu Y, Ma Q, Liu H, Guo Z. Public attitudes and influencing factors toward COVID-19 vaccination for adolescents/children: a scoping review. Public Health. 2022 Apr;205:169-181.	Systematic review
**20**	Garbin AJÍ, Chiba EK, Garbin CAS, Chiba FY, Moimaz SAS, Saliba TA. Systematic review: Impact of parental decision on paediatric COVID-19 vaccination. Child Care Health Dev. 2023 Sep;49(5):787-799.	Systematic review
**21**	Alsuwaidi AR, Hammad HAA, Elbarazi I, Sheek-Hussein M. Vaccine hesitancy within the Muslim community: Islamic faith and public health perspectives. Hum Vaccin Immunother. 2023 Dec 31;19(1):2190716. Epub 2023 Mar 13.	Review article<br>Epub 2023 Mar 13.
**22**	Abu El Kheir-Mataria W, Saleh BM, El-Fawal H, Chun S. COVID-19 vaccine hesitancy among parents in Low- and Middle-Income Countries: A meta-analysis. Front Public Health. 2023 Feb 15;11:1078009.	Meta-Analysis
**23**	Alsalloum MA, Garwan YM, Jose J, Thabit AK, Baghdady N. COVID-19 vaccine hesitancy and acceptance among the public in the Gulf Cooperation Council countries: A review of the literature. Hum Vaccin Immunother. 2022 Nov 30;18(6):2091898.	Review of the literature
**24**	Sallam M. COVID-19 Vaccine Hesitancy Worldwide: A Concise Systematic Review of Vaccine Acceptance Rates. Vaccines (Basel). 2021 Feb 16;9(2):160.	Systematic review
**25**	Wen J, Du X, Li A, Zhang S, Shen S, Zhang Z, et al. Dilemmas and options for COVID-19 vaccination in children. Ital J Pediatr. 2023 Aug 25;49(1):103.	Review of the literature
**26**	Ma Y, Ren J, Zheng Y, Cai D, Li S, Li Y. Chinese parents' willingness to vaccinate their children against COVID-19: A systematic review and meta-analysis. Front Public Health. 2022 Dec 15;10:1087295.	Systematic Review and Meta-Analysis
**27**	Limbu YB, Gautam RK, Zhou W. Predicting Vaccination Intention against COVID-19 Using Theory of Planned Behavior: A Systematic Review and Meta-Analysis. Vaccines (Basel). 2022 Nov 26;10(12):2026.	Systematic Review and Meta-Analysis
**28**	Cénat JM, Noorishad PG, Moshirian Farahi SMM, Darius WP, Mesbahi El Aouame A, Onesi O, et al. Prevalence and factors related to COVID-19 vaccine hesitancy and unwillingness in Canada: A systematic review and meta-analysis. J Med Virol. 2023 Jan;95(1):e28156.	Systematic Review and Meta-Analysis
**29**	Wang Z, Chen S, Fang Y. Parental Willingness and Associated Factors of Pediatric Vaccination in the Era of COVID-19 Pandemic: A Systematic Review and Meta-Analysis. Vaccines (Basel). 2022 Sep 2;10(9):1453.	Systematic Review and Meta-Analysis
**30**	Kazeminia M, Afshar ZM, Rajati M, Saeedi A, Rajati F. Evaluation of the Acceptance Rate of Covid-19 Vaccine and its Associated Factors: A Systematic Review and Meta-analysis. J Prev (2022). 2022 Aug;43(4):421-467.	Systematic Review and Meta-Analysis
**31**	Galal B, Lazieh S, Al-Ali S, Khoshnood K. Assessing vaccine hesitancy in Arab countries in the Middle East and North Africa (MENA) region: a scoping review protocol. BMJ Open. 2022 Feb 11;12(2):e045348.	Scoping review
**32**	Roy DN, Biswas M, Islam E, Azam MS. Potential factors influencing COVID-19 vaccine acceptance and hesitancy: A systematic review. PLoS One. 2022 Mar 23;17(3):e0265496.	Systematic review
**33**	Biswas MR, Alzubaidi MS, Shah U, Abd-Alrazaq AA, Shah Z. A Scoping Review to Find Out Worldwide COVID-19 Vaccine Hesitancy and Its Underlying Determinants. Vaccines (Basel). 2021 Oct 25;9(11):1243.	Scoping review
**34**	She J, Liu L, Liu W. Providing children with COVID-19 vaccinations is challenging due to lack of data and wide-ranging parental acceptance. Acta Paediatr. 2022 Jan;111(1):35-44.	Systematic review
**35**	Joshi A, Kaur M, Kaur R, Grover A, Nash D, El-Mohandes A. Predictors of COVID-19 Vaccine Acceptance, Intention, and Hesitancy: A Scoping Review. Front Public Health. 2021 Aug 13;9:698111.	Scoping review

Non-eligible Articles/ Records excluded because studies are not free full-text (n=21)

Serial	Article	Remarks
1	Alharbi HS. Review: Factors influencing parents' decisions to vaccinate children against COVID-19. Vaccine. 2023 Oct 13;41(43):6419-6425.	
2	Lu JG. Two large-scale global studies on COVID-19 vaccine hesitancy over time: Culture, uncertainty avoidance, and vaccine side-effect concerns. J Pers Soc Psychol. 2023 Apr;124(4):683-706.	
3	Short MB, Marek RJ, Knight CF, Kusters IS. Understanding factors associated with intent to receive the COVID-19 vaccine. Fam Syst Health. 2022 Jun;40(2):160-170.	
4	Stettner NM, Lavelle EN, Cafferty P. Who decides? Consent for healthcare decisions of minors in the United States. Curr Opin Pediatr. 2023 Apr 1;35(2):275-280.	
5	John SD. How low can you go? Justified hesitancy and the ethics of childhood vaccination against COVID-19. J Med Ethics. 2022 Dec;48(12):1006-1009.	
6	Yeskendir A, Gusmanov A, Zhussupov B. Parental attitudes, beliefs and behaviors toward childhood and COVID-19 vaccines: A countrywide survey conducted in Kazakhstan examining vaccine refusal and hesitancy. Vaccine. 2023 Oct 20;41(44):6548-6557.	
7	Yigit M, Ozkaya-Parlakay A, Senel E. Evaluation of COVID-19 Vaccine Refusal in Parents. Pediatr Infect Dis J. 2021 Apr 1;40(4):e134-e136.	
8	Alemán-Reyes AG, Díaz-Rivera E, Rodríguez-Quiñones AJ, Molina-Pérez XS, Oquendo-Claudio GI, Vega A, et al. Correlation between Parental Vaccine Hesitancy, Socio-demographic Factors, and Novel SARS-CoV-2 Vaccination in Puerto Rico. P R Health Sci J. 2022 Dec;41(4):185-191.	
9	Kilci C, Fettah A, Çapkinoğlu E, Kurucu B, Ünüvar Gök Ş, Yeşil Ş, et al. Evaluation of the Attitude of Parents in Pediatric Oncology Towards COVID-19 Vaccine. J Pediatr Hematol Oncol. 2023 Jan 1;45(1):e14-e16.	
10	Duran S, Duran R, Acunaş B, Şahin EM. Changes in parents' attitudes towards childhood vaccines during COVID-19 pandemic. Pediatr Int. 2023 Jan-Dec;65(1):e15520.	
11	Lazarus JV, Wyka K, White TM, Picchio CA, Gostin LO, Larson HJ, et al. A survey of COVID-19 vaccine acceptance across 23 countries in 2022. Nat Med. 2023 Feb;29(2):366-375.	
12	Husted M, Gibbons A, Cheung WY, Keating S. COVID-19 vaccination hesitancy in adults in the United Kingdom: Barriers and facilitators to uptake. Health Psychol. 2023 Aug;42(8):584-592.	
13	Huynh G, Nguyen HTN, Van Tran K, Le An P, Tran TD. Determinants of COVID-19 vaccine hesitancy among parents in Ho Chi Minh City, Vietnam. Postgrad Med. 2022 Apr;134(3):303-308.	
14	Kaufman J, Steffens MS, Hoq M, King C, Marques MD, Mao K, Bullivant B, et al. Effect of persuasive messaging about COVID-19 vaccines for 5- to 11-year-old children on parent intention to vaccinate. J Paediatr Child Health. 2023 Apr;59(4):686-693.	
15	Aggarwal S, Madaan P, Sharma M. Vaccine Hesitancy Among Parents of Children With Neurodevelopmental Disabilities: A Possible Threat to COVID-19 Vaccine Coverage. J Child Neurol. 2022 Jan;37(1):99.	
16	Olick RS, Yang YT, Shaw J. When Adolescents Disagree with Their Vaccine-Hesitant Parents about COVID-19 Vaccination. J Clin Ethics. 2023 Summer;34(2):158-168	
17	Rhodes ME, Sundstrom B, Ritter E, McKeever BW, McKeever R. Preparing for A COVID-19 Vaccine: A Mixed Methods Study of Vaccine Hesitant Parents. J Health Commun. 2020 Oct 2;25(10):831-837.	
18	Dao TL, Vu Thi H, Gautret P, Al-Tawfiq JA, Nguyen TL, Chu DT, Hoang VT. Willingness and attitudes of parents towards COVID-19 vaccines for children in Vietnam. J Commun Healthc. 2023 Mar;16(1):75-82.	
19	Yigit M, Ozkaya-Parlakay A, Senel E. Evaluation of COVID-19 Vaccine Refusal in Parents. Pediatr Infect Dis J. 2021 Apr 1;40(4):e134-e136.	
20	Bourque SL, Weikel BW, Palmer C, Cataldi JR, Blackwell S, Hwang SS. Prevalence and Predictors of Pediatric COVID-19 Vaccine Acceptance. Am J Perinatol. 2023 Jan;40(1):106-114.	
21	McNally VV, Bernstein HH. The Effect of the COVID-19 Pandemic on Childhood Immunizations: Ways to Strengthen Routine Vaccination. Pediatr Ann. 2020 Dec 1;49(12):e516-e522.	

Non-eligible Articles/ Records excluded because studies are irrelevant or with different research question (n=47)

Serial	Article	Remarks
**1**	Al Naam YA, Elsafi SH, Alkharraz ZS, Almaqati TN, Alomar AM, et al. Factors related to COVID-19 vaccine hesitancy in Saudi Arabia. Public Health Pract (Oxf). 2022 Jun;3:100258.	
**2**	Cag Y, Al Madadha ME, Ankarali H, Cag Y, Demir Onder K, Seremet-Keskin A, et al. Vaccine hesitancy and refusal among parents: An international ID-IRI survey. J Infect Dev Ctries. 2022 Jun 30;16(6):1081-1088.	Pooled data without data specific to KSA
**3**	Khatatbeh M, Albalas S, Khatatbeh H, Momani W, Melhem O, Al Omari O et al. Children's rates of COVID-19 vaccination as reported by parents, vaccine hesitancy, and determinants of COVID-19 vaccine uptake among children: a multi-country study from the Eastern Mediterranean Region. BMC Public Health. 2022 Jul 18;22(1):1375.	Pooled data without data specific to KSA
**4**	Alzahrani AA, Alghamdi AN. Vaccine Hesitancy Among Parents and Its Determinants During the Era of COVID-19 in Taif City, Saudi Arabia. Cureus. 2023 Jun 14;15(6):e40404.	
**5**	Meraya AM, Salami RM, Alqahtani SS, Madkhali OA, Hijri AM, Qassadi FA, Albarrati AM. COVID-19 Vaccines and Restrictions: Concerns and Opinions among Individuals in Saudi Arabia. Healthcare (Basel). 2022 Apr 28;10(5):816.	
**6**	Othman SS, Alsuwaidi A, Aseel R, Alotaibi R, Bablgoom R, Alsulami G, et al. Association between social media use and the acceptance of COVID-19 vaccination among the general population in Saudi Arabia - a cross-sectional study. BMC Public Health. 2022 Feb 21;22(1):375.	
**7**	Khalafalla HE, Tumambeng MZ, Halawi MHA, Masmali EMA, Tashari TBM, Arishi FHA, et al. COVID-19 Vaccine Hesitancy Prevalence and Predictors among the Students of Jazan University, Saudi Arabia Using the Health Belief Model: A Cross-Sectional Study. Vaccines (Basel). 2022 Feb 14;10(2):289.	
**8**	Marzo RR, Sami W, Alam MZ, Acharya S, Jermsittiparsert K, Songwathana K, et al. Hesitancy in COVID-19 vaccine uptake and its associated factors among the general adult population: a cross-sectional study in six Southeast Asian countries. Trop Med Health. 2022 Jan 5;50(1):4.	
**9**	Mohammed AH, Hassan BAR, Wayyes AM, Gadhban AQ, Blebil A, Alhija SA, et al. Parental health beliefs, intention, and strategies about covid-19 vaccine for their children: A cross-sectional analysis from five Arab countries in the Middle East. Vaccine. 2022 Oct 26;40(45):6549-6557.	No data from KSA
**10**	Ghamri RA, Othman SS, Alhiniah MH, Alelyani RH, Badawi AM, Alshahrani AA. Acceptance of COVID-19 Vaccine and Associated Factors Among Pregnant Women in Saudi Arabia. Patient Prefer Adherence. 2022 Apr 2;16:861-873.	
**11**	Makki S, Siddiqua A, Wahab S, Siddiqui S, Faheemuddin M, Abobaker I, et al. Beliefs and Barriers to COVID-19 Vaccine Acceptance in Three Countries with Different Human Development Index (HDI) Scores: A Comparative Study. Patient Prefer Adherence. 2022 Dec 13;16:3257-3265.	
**12**	AlSaeed AA, Rabbani U. Explaining COVID-19 Vaccine Rejection Using Social Cognitive Theory in Qassim, Saudi Arabia. Vaccines (Basel). 2021 Nov 9;9(11):1304.	
**13**	Kaadan MI, Abdulkarim J, Chaar M, Zayegh O, Keblawi MA. Determinants of COVID-19 vaccine acceptance in the Arab world: a cross-sectional study. Glob Health Res Policy. 2021 Jul 12;6(1):23.	
**14**	Odeh NB, Sriwi TH, Arbili LM, Arabi TZ, Sabbah BN, Alkodaymi MS. Describing the Myths and Misconceptions Regarding COVID-19 Vaccines Among the Population of the Kingdom of Saudi Arabia. Cureus. 2022 Jun 14;14(6):e25932.	
**15**	Alharbi I, Alharthi R, Aljabri S, Alzhrani R, Alzahrani L, Albagami S. Seasonal Influenza Vaccination Among Saudi Children: Parental Barriers and Willingness to Vaccinate Their Children in the Makkah Region. Cureus. 2023 May 11;15(5):e38878.	
**16**	Khatatbeh M, Albalas S, Khatatbeh H, Momani W, Melhem O, Al Omari O, et al. Children's rates of COVID-19 vaccination as reported by parents, vaccine hesitancy, and determinants of COVID-19 vaccine uptake among children: a multi-country study from the Eastern Mediterranean Region. BMC Public Health. 2022 Jul 18;22(1):1375.	
**17**	Bono SA, Siau CS, Chen WS, Low WY, Faria de Moura Villela E, Pengpid S, Hasan MT, Sessou P, Ditekemena JD, Amodan BO, Hosseinipour MC, Dolo H, Siewe Fodjo JN, Colebunders R. Adults' Acceptance of COVID-19 Vaccine for Children in Selected Lower- and Middle-Income Countries. Vaccines (Basel). 2021 Dec 22;10(1):11.	
**18**	Aldakhil H, Albedah N, Alturaiki N, Alajlan R, Abusalih H. Vaccine hesitancy towards childhood immunizations as a predictor of mothers' intention to vaccinate their children against COVID-19 in Saudi Arabia. J Infect Public Health. 2021 Oct;14(10):1497-1504.	

**19**	Almojaibel A, Ansari K, Alzahrani Y, Alquaimi M, Farooqi F, Alqurashi Y. COVID-19 vaccine hesitancy in the Saudi Arabian population. J Med Life. 2023 Jan;16(1):101-109.	
**20**	Yahia AIO, Alshahrani AM, Alsulmi WGH, Alqarni MMM, Abdulrahim TKA, Heba WFH, et al. Determinants of COVID-19 vaccine acceptance and hesitancy: a cross-sectional study in Saudi Arabia. Hum Vaccin Immunother. 2021 Nov 2;17(11):4015-4020.	
**21**	Alfatease A, Alqahtani AM, Orayj K, Alshahrani SM. The Impact of Social Media on the Acceptance of the COVID-19 Vaccine: A Cross-Sectional Study from Saudi Arabia. Patient Prefer Adherence. 2021 Nov 30;15:2673-2681.	
**22**	Gray J, AlAnazi AA, AlSumait F, Abu-Shaheen A, Bashir MS, Al Sheef M. What motivated residents of Saudi Arabia to receive the COVID-19 vaccine? Front Public Health. 2023 Feb 7;11:1065157.	
**23**	Alrowaily M, Alkhathlan T, Alaql A, Almesned I, Alrowaily H, Alayed N, et al. Assessment of concerns about vaccination among recovered COVID-19 patients in Saudi Arabia. East Mediterr Health J. 2023 Apr 27;29(4):276-284.	
**24**	Munshi A, Alhouthali A, Munshi E, Mujalled MK, Alqalayta L, Zahed H, et al. COVID-19 Vaccination Knowledge and Attitude Among the General Population in Jeddah, Saudi Arabia. Cureus. 2023 Aug 4;15(8):e42951.	
**25**	Fadhel FH. Vaccine hesitancy and acceptance: an examination of predictive factors in COVID-19 vaccination in Saudi Arabia. Health Promot Int. 2023 Aug 1;38(4):daab209.	
**26**	Abdalla SM, Mohamed EY, Elsabagh HM, Ahmad MS, Shaik RA, Mehta V, et al. COVID-19 Vaccine Hesitancy among the General Population: A Cross-Sectional Study. Vaccines (Basel). 2023 Jun 20;11(6):1125.	
**27**	Peters MDJ. Addressing vaccine hesitancy and resistance for COVID-19 vaccines. Int J Nurs Stud. 2022 Jul;131:104241.	
**28**	Alghamdi AA, Alghamdi HA. Knowledge, Attitude, and Practice of Vaccination Among Parents in Jeddah City, Saudi Arabia. Cureus. 2023 Jul 11;15(7):e41721.	
**29**	Qashqari FSI, Dahlawi M, Assaggaf HM, Alsafi R, Gari A, Abudawood A, et al. Effect of the COVID-19 Vaccine on the Menstrual Cycle among Females in Saudi Arabia. Ethiop J Health Sci. 2022 Nov;32(6):1083-1092.	
**30**	Alghofaili MA, Aljuaid SO, Alqahtani N, Alghufaili M, Abd-Ellatif EE. Factors Contributing to the Delayed Vaccination Among Children in Riyadh City, Saudi Arabia: A Cross-Sectional Study. Cureus. 2023 Aug 9;15(8):e43188.	
**31**	Baghdadi LR, Hassounah MM, Younis A, Al Suwaidan HI, Al Khalifah R. Caregivers' Sources of Information About Immunization as Predictors of Delayed Childhood Vaccinations in Saudi Arabia During the COVID-19 Pandemic: A Cross-Sectional Questionnaire Study. Risk Manag Healthc Policy. 2021 Aug 24;14:3541-3550.	
**32**	Abullais SS, Arora S, Parveen S, Mahmood SE, Baba SM, Khalid I, et al. Perceptions, motivation factors, and barriers to a COVID-19 booster immunization in a subpopulation of KSA: A cross-sectional study. Medicine (Baltimore). 2022 Nov 25;101(47):e31669.	
**33**	Abdalla SM, Ahmad MS, Saleem Al-Baradie NR, Mohammed Alshuwaish LA, Ahmad Al-Issa RA, Suwaylih Alrashidi SN. Assessment of parent knowledge and perception towards the importance of child immunization in Sudair region, Saudi Arabia. Eur Rev Med Pharmacol Sci. 2022 Mar;26(6):1803-1808.	
**34**	Alshammari SZ, AlFayyad I, Altannir Y, Al-Tannir M. Parental Awareness and Attitude about Childhood Immunization in Riyadh, Saudi Arabia: A Cross-Sectional Study. Int J Environ Res Public Health. 2021 Aug 10;18(16):8455.	
**35**	Alfieri NL, Kusma JD, Heard-Garris N, Davis MM, Golbeck E, Barrera L, et al. Parental COVID-19 vaccine hesitancy for children: vulnerability in an urban hotspot. BMC Public Health. 2021 Sep 13;21(1):1662.	
**36**	Abushouk A, Ahmed ME, Althagafi Z, Almehmadi A, Alasmari S, Alenezi F, et al. Knowledge, attitude, and practice toward seasonal influenza vaccine during the COVID-19 pandemic among students at King Saud bin Abdulaziz University for Health Sciences-Jeddah, Saudi Arabia. J Educ Health Promot. 2023 Jan 31;12:17.	
**37**	Alzahrani SH, Baig M, Alrabia MW, Algethami MR, Alhamdan MM, Alhakamy NA, et al. Attitudes toward the SARS-CoV-2 Vaccine: Results from the Saudi Residents' Intention to Get Vaccinated against COVID-19 (SRIGVAC) Study. Vaccines (Basel). 2021 Jul 18;9(7):798.	
**38**	Khan A, Alsofayan Y, Alahmari A, Alowais J, Algwizani A, Alserehi H, Assiri A, Jokhdar H. COVID-19 in Saudi Arabia: the national health response. East Mediterr Health J. 2021 Dec 1;27(11):1114-1124.	

**39**	ElSayed DA, Bou Raad E, Bekhit SA, Sallam M, Ibrahim NM, Soliman S, et al. Validation and Cultural Adaptation of the Parent Attitudes about Childhood Vaccines (PACV) Questionnaire in Arabic Language Widely Spoken in a Region with a High Prevalence of COVID-19 Vaccine Hesitancy. Trop Med Infect Dis. 2022 Sep 8;7(9):234.	
**40**	Hobani F, Alhalal E. Factors related to parents' adherence to childhood immunization. BMC Public Health. 2022 Apr 25;22(1):819.	
**41**	Mubarak S, A'aqoulah A, AlGhawrie H, Albalas S, Innab N. Assessing the acceptability of COVID-19 vaccine and its booster dose. Immun Inflamm Dis. 2023 Sep;11(9):e950.	
**42**	Adam M, Gameraddin M, Alelyani M, Alshahrani MY, Gareeballah A, Ahmad I, et al. Evaluation of Post-Vaccination Symptoms of Two Common COVID-19 Vaccines Used in Abha, Aseer Region, Kingdom of Saudi Arabia. Patient Prefer Adherence. 2021 Sep 7;15:1963-1970.	
**43**	Aldali J, Meo SA, Al-Khlaiwi T. Adverse Effects of Pfizer (BioNTech), Oxford-AstraZeneca (ChAdOx1 CoV-19), and Moderna COVID-19 Vaccines among the Adult Population in Saudi Arabia: A Cross-Sectional Study. Vaccines (Basel). 2023 Jan 20;11(2):231.	
**44**	Alrajeh AM, Daghash H, Buanz SF, Altharman HA, Belal S. COVID-19 Vaccine Hesitancy Among the Adult Population in Saudi Arabia. Cureus. 2021 Dec 6;13(12):e20197.	
**45**	Al-Zalfawi SM, Rabbani SI, Asdaq SMB, Alamri AS, Alsanie WF, Alhomrani M, Mohzari Y, Alrashed AA, AlRifdah AH, Almagrabe T. Public Knowledge, Attitude, and Perception towards COVID-19 Vaccination in Saudi Arabia. Int J Environ Res Public Health. 2021 Sep 25;18(19):10081.	
**46**	Alzamil Y, Almeshari M, Alyahyawi A, Abanomy A, Al-Thomali AW, Alshomar B, et al. Knowledge, attitude, and practice of the Saudi population toward COVID-19 vaccination: A cross-sectional study. Medicine (Baltimore). 2023 Oct 13;102(41):e35360.	
**47**	Alam MM, Melhim LKB, Ahmad MT, Jemmali M. Public Attitude Towards COVID-19 Vaccination: Validation of COVID-Vaccination Attitude Scale (C-VAS). J Multidiscip Healthc. 2022 Apr 29;15:941-954.	

Non-eligible Articles/ Records excluded because studies are published in language other than English (n=13)

Serial	Article	Remarks
**1**	Brandstetter S, Pawellek M, Böhmer MM, Köninger A, Melter M, Kabesch M, et al. COVID-19-Impfintention von Eltern bezogen auf ihre Kinder [Parental intention to vaccinate their children against COVID-19]. Bundesgesundheitsblatt Gesundheitsforschung Gesundheitsschutz. 2022 Dec;65(12):1281-1288.	Additionally, study from Germany
**2**	Wang SY, Pan XJ, Deng X, Zhang HJ, Chen ZP. [An exploratory framework of vaccine hesitancy monitoring and early warning system in China]. Zhonghua Yu Fang Yi Xue Za Zhi. 2021 Aug 6;55(8):925-930.	Additionally, study with different research question from China
**3**	Caballero P, Astray J, Domínguez Á, Godoy P, Barrabeig I, Castilla J, et al. Validation of the questionnaire on vaccines and hesitancy to be vaccinated in the Spanish Society of Epidemiology. Gac Sanit. 2023 Oct 9;37:102329.	Additionally, study from Spain with different research question
**4**	Choi IS, Kim EA. [Factors Influencing the COVID-19 Vaccination Intentions in Parents for Their Children Aged 5~11: Korea, April 2022]. J Korean Acad Nurs. 2023 Apr;53(2):208-221.	Additionally, study from Korea
**5**	Omari HE, Chahlaoui A, El Ouali Lalami A. Intention to vaccinate under-12 children against COVID-19 by parents in the Meknes Prefecture of Morocco. East Mediterr Health J. 2022 Nov 30;28(11):835-839.	Additionally, study from Morocco
**6**	Caycho-Rodríguez T, Gallegos M, Valencia PD, Vilca LW. ¿Cuánto apoyan los peruanos las creencias de conspiración sobre las vacunas contra la COVID-19? [How much do Peruvians support conspiracy beliefs about COVID-19 vaccines?]. Aten Primaria. 2022 May;54(5):102318. Spanish.	Additionally, irrelevant study from Peru
**7**	Picazo JJ. Vacuna frente al COVID-19 [Vaccine against COVID-19]. Rev Esp Quimioter. 2021 Dec;34(6):559-598. Spanish.	Additionally, study with different research question
**8**	de Andrés Sánchez J, Arias-Oliva M, Pelegrín-Borondo J, Lima Rua O. Factores explicativos de la aceptación de la vacuna para el SARS-CoV-2 desde la perspectiva del comportamiento del consumidor [Explanatory factors on the acceptance of SARS-CoV-2 vaccine from consumer's behaviour perspective.]. Rev Esp Salud Publica. 2021 Jul 28;95:e202107101. Spanish.	Additionally, study with different research question
**9**	Monschein T, Zrzavy T, Löbermann M, Winkelmann A, Berger T, Rommer P, et al. Die Corona-Pandemie und Multiple Sklerose: Impfungen und deren Implikationen für Patienten – Teil 2: Impfstofftechnologien [The corona pandemic and multiple sclerosis: vaccinations and their implications for patients-Part 2: vaccine technologies]. Nervenarzt. 2021 Dec;92(12):1283-1292. German.	Additionally, study with different research question
**10**	Agut H. Covid-19 - Le miracle vaccinal doit inclure rigueur et prudence [COVID-19 - The vaccine miracle must include strictness and caution]. Virologie (Montrouge). 2021 Jun 1;25(3):141-147. French.	Additionally, study with different research question
**11**	Moraga-Llop F. Vacunación frente a la COVID-19 en los adolescentes. Una realidad [Vaccination against COVID-19 in adolescents. A reality]. Vacunas. 2021 Sep-Dec;22(3):135-137. Spanish.	Additionally, study with different research question
**12**	Gonzales-Zamora JA, Soriano-Moreno DR, Soriano AN, Ponce-Rosas L, De-Los-Rios-Pinto A, et al. Percepciones e intención de los padres de vacunar a sus hijos bajo 12 años de edad contra la COVID-19: estudio transversal en Perú [Parents' perceptions and intention to vaccinate their children under 12 years of age against COVID-19: a cross-sectional study in Peru]. Rev Chilena Infectol. 2022 Jun;39(3):273-286. Spanish.	Additionally, study from Peru
**13**	Salvador PTCO, Alves KYA, Carvalho KRS, Nehab MF, Camacho KG, Reis AT, et al. Inquérito online sobre os motivos para hesitação vacinal contra a COVID-19 em crianças e adolescentes do Brasil [Online survey on the reasons for vaccine hesitancy against COVID-19 in children and adolescents in Brazil]. Cad Saude Publica. 2023 Oct 13;39(10):e00159122. Portuguese.	Additionally, study from Brazil

Studies with only subgroup-specific samples of either participating parents or their children (n=29)

Serial	Article	Remarks
**1**	Aldossari KK, Alharbi MB, Alkahtani SM, Alrowaily TZ, Alshaikhi AM, Twair AA. COVID-19 vaccine hesitancy among patients with diabetes in Saudi Arabia. Diabetes Metab Syndr. 2021 Sep-Oct;15(5):102271.	
**2**	Al Saad AJ, Alhassan GM, Albedaiwi MS, Alqattan FF, Aleisa FA, Alabdulmuhsin HW. Acceptance of COVID-19 vaccination among parents of children with autism and other neurodevelopmental disorders in Saudi Arabia: a cross-sectional study. BMC Public Health. 2023 Jun 26;23(1):1235.	
**3**	Khoodoruth MAS, Khoodoruth WNC, Ramadan AAM, Johnson B, Gulistan S, Deluvio RBC, et al. Evaluating COVID-19 vaccination intentions and vaccine hesitancy among parents of children with autism spectrum disorder. Sci Rep. 2023 May 5;13(1):7353.	
**4**	Costantino A, Morlacchi L, Donato MF, Gramegna A, Farina E, Dibenedetto C, et al. Hesitancy toward the Full COVID-19 Vaccination among Kidney, Liver and Lung Transplant Recipients in Italy. Vaccines (Basel). 2022 Nov 10;10(11):1899.	
**5**	Tsai CS, Hsiao RC, Chen YM, Yen CF. Factors Related to Caregiver Intentions to Vaccinate Their Children with Attention-Deficit/Hyperactivity Disorder against COVID-19 in Taiwan. Vaccines (Basel). 2021 Sep 2;9(9):983.	
**6**	Barry M, Temsah MH, Aljamaan F, Saddik B, Al-Eyadhy A, Alenezi S, et al. COVID-19 vaccine uptake among healthcare workers in the fourth country to authorize BNT162b2 during the first month of rollout. Vaccine. 2021 Sep 24;39(40):5762-5768.	
**7**	Alghamdi AA, Aldosari MS, Alsaeed RA. Acceptance and barriers of COVID-19 vaccination among people with chronic diseases in Saudi Arabia. J Infect Dev Ctries. 2021 Nov 30;15(11):1646-1652.	
**8**	Skeens MA, Hill K, Olsavsky A, Buff K, Stevens J, Akard TF, et al. Factors affecting COVID-19 vaccine hesitancy in parents of children with cancer. Pediatr Blood Cancer. 2022 Jun;69(6):e29707.	
**9**	Elkhadry SW, Salem TAEH, Elshabrawy A, Goda SS, Bahwashy HAA, Youssef N, Hussein M, Ghazy RM. COVID-19 Vaccine Hesitancy among Parents of Children with Chronic Liver Diseases. Vaccines (Basel). 2022 Dec 7;10(12):2094.	
**10**	Alobaidi S, Alsolami E, Sherif A, Almahdy M, Elmonier R, Alobaidi WY, et al. COVID-19 Booster Vaccine Hesitancy among Hemodialysis Patients in Saudi Arabia Using the Health Belief Model: A Multi-Centre Experience. Vaccines (Basel). 2022 Dec 31;11(1):95.	
**11**	Al-Hanawi MK, Ahmad K, Haque R, Keramat SA. Willingness to receive COVID-19 vaccination among adults with chronic diseases in the Kingdom of Saudi Arabia. J Infect Public Health. 2021 Oct;14(10):1489-1496.	
**12**	Almalki MJ, Alotaibi AA, Alabdali SH, Zaalah AA, Maghfuri MW, Qirati NH, et al. Acceptability of the COVID-19 Vaccine and Its Determinants among University Students in Saudi Arabia: A Cross-Sectional Study. Vaccines (Basel). 2021 Aug 25;9(9):943.	
**13**	Wang CS, Doma R, Westbrook AL, Johnson J, Anderson EJ, Greenbaum LA, et al. Vaccine Attitudes and COVID-19 Vaccine Intention Among Parents of Children With Kidney Disease or Primary Hypertension. Am J Kidney Dis. 2023 Jan;81(1):25-35.e1.	
**14**	Robert AA, Al Saeed A, Al Dawish MA. COVID-19 among people with diabetes mellitus in Saudi Arabia: Current situation and new perspectives. Diabetes Metab Syndr. 2021 Sep-Oct;15(5):102231.	
**15**	Akgün Ö, Kayaalp GK, Demirkan FG, Çakmak F, Tanatar A, Guliyeva V, et al. Exploring the attitudes, concerns, and knowledge regarding COVID-19 vaccine by the parents of children with rheumatic disease: Cross-sectional online survey. Vaccine. 2022 Mar 15;40(12):1829-1836.	
**16**	Alfosail EK, Alghamdi M. Perception and Acceptance of COVID-19 Vaccine Among Healthcare Workers in Jeddah, Saudi Arabia. Cureus. 2023 Mar 1;15(3):e35673.	
**17**	Omer I, Alhuzali A, Aletani T, Althagafi Z, Ghulam E, Awadh A. Vaccine Hesitancy among Medical Students at a Tertiary Hospital-Affiliated Medical School. Healthcare (Basel). 2023 Feb 5;11(4):461.	

**18**	Arif SI, Aldukhail AM, Albaqami MD, Silvano RC, Titi MA, Arif BI, et al. Predictors of healthcare workers' intention to vaccinate against COVID-19: A cross sectional study from Saudi Arabia. Saudi J Biol Sci. 2022 Apr;29(4):2314-2322.	
**19**	Althaqafi A, Munshi A, Mujalled MK, Munshi E, Alhouthali A, Alqalayta L, et al. COVID-19 Vaccine Knowledge and Attitude Among Healthcare Workers in Jeddah, Saudi Arabia. Cureus. 2023 Jun 28;15(6):e41070.	
**20**	Mohamed R, White TM, Lazarus JV, Salem A, Kaki R, Marrakchi W, et al. COVID-19 vaccine acceptance and associated factors among people living with HIV in the Middle East and North Africa region. South Afr J HIV Med. 2022 Aug 24;23(1):1391.	
**21**	Ghazy RM, Sallam M, Fadl N, Bouraad E, Youssef N, Ghoneim OSA. Attitude of Parents of Children with Cerebral Palsy Towards COVID-19 Vaccination. Int J Environ Res Public Health. 2023 Jan 20;20(3):1909.	
**22**	Qattan AMN, Alshareef N, Alsharqi O, Al Rahahleh N, Chirwa GC, Al-Hanawi MK. Acceptability of a COVID-19 Vaccine Among Healthcare Workers in the Kingdom of Saudi Arabia. Front Med (Lausanne). 2021 Mar 1;8:644300.	
**23**	Bonsu NEM, Mire SS, Sahni LC, Berry LN, Dowell LR, Minard CG, et al. Understanding Vaccine Hesitancy Among Parents of Children With Autism Spectrum Disorder and Parents of Children With Non-Autism Developmental Delays. J Child Neurol. 2021 Sep;36(10):911-918.	
**24**	Maqsood MB, Islam MA, Al Qarni A, Nisa ZU, Ishaqui AA, Alharbi NK, et al. Assessment of COVID-19 Vaccine Acceptance and Reluctance Among Staff Working in Public Healthcare Settings of Saudi Arabia: A Multicenter Study. Front Public Health. 2022 May 30;10:847282.	
**25**	Mubarak AS, Baabbad AS, Almalki NA, Alrbaiai GT, Alsufyani GA, Kabrah DK. Beliefs, barriers, and acceptance associated with COVID-19 vaccination among Taif University students in Saudi Arabia. J Family Med Prim Care. 2022 Jan;11(1):224-232.	
**26**	Homaira N, Chan M, Owens L, Thomsen A, Gray M, Chuang S, et al. Parent/carers' opinions about COVID-19 vaccination for children with chronic lung diseases. Health Sci Rep. 2021 Oct 1;4(4):e410.	
**27**	Altulaihi BA, Alharbi KG, Alaboodi TA, Alkanhal HM, Alobaid MM, Aldraimly MA. Factors and Determinants for Uptake of COVID-19 Vaccine in a Medical University in Riyadh, Saudi Arabia. Cureus. 2021 Sep 6;13(9):e17768.	
**28**	Aldosary AH, Alayed GH. Willingness to vaccinate against Novel COVID-19 and contributing factors for the acceptance among nurses in Qassim, Saudi Arabia. Eur Rev Med Pharmacol Sci. 2021 Oct;25(20):6386-6396.	
**29**	Alkeridy WA, Alquaydheb H, Almuhaidib S, Sindi NA, Aljasser A, Kushner Kow J, et al. Determinants of Vaccine Hesitancy among Home Health Care Service Recipients in Saudi Arabia. Vaccines (Basel). 2023 Aug 31;11(9):1436.	
