# Supplementary material for: Navigating the shots: Parental willingness to immunize their children with COVID-19 vaccines in Saudi Arabia explored through a systematic review and meta-analysis
Source: PLoS One. 2025 Jan 27;20(1):e0317983. doi: 10.1371/journal.pone.0317983 (PMC11771943; doi:10.1371/journal.pone.0317983)
Supplement: S3 Table — (PDF) [file pone.0317983.s004.pdf]

Table S3. Determinants (factors/predictors) of parental willingness to vaccinate their children against COVID-19

Determinant (Factors/Predictors)	Number of studies With such determinant	Individual studies by author name	Number of parents willing to vaccinate their children N %	Willingness rate in relation to each determinant in each study Number %	Total willing parents N %	Refusal rate in relation to each determinant in each study Number %	Total refusing parents N %	OR (95% CI) for willingness	p value
**Age**	10	Almalki OS et al	1577/4135 38.1						
Young parents below 40y and willing				1061/1577 67.3	2791/4654 60.0	1784/2558 69.7	3267/4955 65.9	1.29 (1.19- 1.40)	< 0.0001
Vs Older parents above 40y and willing				516/1577 32.7	1863/4654 40.0	774/2558 30.3	1688/4955 34.1		
		Ennaceur S and Al-Mohaithef	167/379 44.0						
Young parents below 40y and willing				143/167 85.6%		186/212 87.7			
Vs Older parents above 40y and willing				24/167 14.4%		26/212 12.3			
		Aedh AI et al	129/464 27.8						
Young parents below 40y and willing				64/129 49.6%		43/72 59.7			
Vs Older parents above 40y and willing				65/129 50.4%		29/72 40.3			
		AlQahtani AM et al	86/528 16.28						
Young parents below 40y and willing				58/86 67.4%		162/203 79.8			
Vs Older parents above 40y and willing				28/86 32.6%		41/203 20.2			
		Almuqbil M et al	291/699 41.6						
Young parents below 40y and willing				211/291 72.5%		337/408 82.6			
Vs Older parents above 40y and willing				80/291 27.5%		71/408 17.4			
		Iqbal MS et al	1352/1507 89.7						
Young parents below 40y and willing				349/727 48%		118/345 34.2			
Vs Older parents above 40y and willing				378/727 52%		227/345 65.8			
		Khatrawi EM and Sayed AA	130/344 37.8						
Young parents below 40y and willing				70/130 53.8		93/170 54.7			
Vs Older parents above 40y and willing				60/130 46.2		77/170 45.3			
		Alalmaei Asiri et al	465/620 75%						
Young parents below 40y and willing				345/465 74%		119/155 76.8			
Vs Older parents above 40y and willing				120/465 26%		36/155 23.2			
		Al-khlaiwi T et al	602/1304 46.1						
Young parents below 40y and willing				180/602 29.9%		140/383 36.6			
Vs Older parents above 40y and willing				422/602 70.1%		243/383 63.4			
Young parents below 40y and willing		Shati AA et al	810/1463 55.4	310/480 64.5		285/449 63.5			
Vs Older parents above 40y and willing				170/480 35.5		164/449 36.5			

**Gender**	13		256/1000 25.6%						
Mothers		Almusbah Z et al		190/256 74%	3188/5189 61.4	250/300 83.3	4264/5802 73.5	1.74 (1.61- 1.89)	< 0.0001
Fathers				66/256 26%	2001/5189 38.6	50/300 16.7	1538/5802 26.5		
		Almalki OS et al							
Mothers				1192/1577 75.6		2159/2558 84.4			
Fathers		Ennaceur S and Al-Mohaithef		385/1577 24.4		399/2558 15.6			
Mothers				76/167 45.5		112/212 52.8			
Fathers				91/167 54.5		100/212 47.2			
		Aedh Al et al							
Mothers			188/444 42.3	46/129 35.7%		29/72 40.3			
Fathers				83/129 64.3%		43/72 59.7			
		Khan YH et al							
Mothers				132/188 70.2		157/256 61.3			
Fathers				56/188 29.8		99/256 38.7			
		AlQahtani AM et al							
Mothers				64/86 74.4		171/203 84.2			
Fathers				22/86 25.6%		32/203 15.8			
		Almuqbil M et al							
Mothers				232/283 82%		349/399 87.5			
Fathers				51/283 18%		50/399 12.5			
		Iqbal MS et al							
Mothers				156/537 29.1		64/435 14.7			
Fathers				381/537 70.9		371/435 85.3			
		Khatrawi EM and							
Mothers		Sayed AA		66/130 50.8		102/170 60.0			
Fathers				64/130 49.2		68/170 40.0			
		Alalmaei Asiri et al							
Mothers			289/500 57.8	191/465 40.9%		74/154 48.1			
Fathers				274/465 59.1		80/154 51.9			
		Almansour A et al							
Mothers				206/289 71.3		183/211 86.7			
Fathers				83/289 28.7%		28/211 13.3			
Mothers		Al-khlaiwi T et al		404/602 67.1%		313/383 81.7			
Fathers				198/602 32.9%		70/383 18.3			
Mothers		Shati AA et al		233/480 48.5		301/449 67.0			
Fathers				247/480 51.5		148/449 33.0			

**Education level**	13	Almalki OS et al			3038/5165 58.8		3432/5939 57.8	1.04 (0.97- 1.13)	p= 0.27
High (University and above)				756/1577 48	2127/5165 41.2	1464/2558 57.2	2507/5939 42.2		
Low (High school and below)				821/1577 52		1094/2558 42.8			
		Al-Rasheedi AT et	198/597 33.2%						
High		al		125/198 (63.1%)		247/341 72.4			
Low				73/198 (36.9%)		94/341 27.6			
		Ennaceur S and							
High		Al-Mohaithef		143/167 85.6		176/212 83.0			
Low				24/167 14.4		36/212 17.0			
		Aedh AI et al							
High				77/129 59.7		48/72 66.7			
Low				52/129 40.3		24/72 33.3			
		Khan YH et al							
High				102/188 54.2		107/256 41.8			
Low				86/188 45.8		149/256 58.2			
		AlQahtani AM et al							
High				70/86 81.4%		171/203 84.2			
Low		Almuqbil M et al		16/86 18.6		32/203 15.8			
High				182/291 62.5%		285/408 69.9			
Low		Iqbal MS et al		109/291 37.5%		123/408 30.1			
High		Khatrawi EM and		74/563 13.1		48/521 9.2			
Low		Sayed AA		489/563 86.9		473/521 90.8			
High		Alalmaei Asiri et al		111/130 85.4		139/170 81.8			
Low				19/130 14.6		31/170 18.2			
High				310/465 66.7%		98/155 63.2			
Low		Almansour A et al		155/465 33.3%		57/155 36.8			
High				232/289 80.3%		180/211 85.3			
Low		Al-khlaiwi T et al		57/289 19.7%		31/211 14.7			
High				505/602 83.9		342/383 89.3			
Low		Shati AA et al		97/602 16.1%		41/383 10.7			
High				351/480 73.1		127/449 28.3			
Low				129/480 26.9		322/449 71.7			

**Children age**<br>Young below 5y<br>5-12y<br><br>Young below 5y<br>5-12y<br><br>Young below 5y<br>5-12y	3	Almusbah Z et al<br><br><br><br>Aedh AI et al<br><br><br><br>AlQahtani AM et al		 156/256 61%<br>100/256 39%<br><br>38/129 29.5<br>91/129 70.5<br><br>14/86 16.3<br>72/86 83.7	 208/471 44.2<br>263/471 55.8	 230/360 63.9<br>130/360 36.1<br><br>16/72 22.2<br>56/72 77.7<br><br>18/203 8.9<br>185/203 91.1	 264/635 41.6<br>371/635 58.4	1.11 (0.87-1.41)	p=0.39
**Nationality**<br>Saudi<br>Non-Saudi<br><br>Saudi<br>Non-Saudi<br><br>Saudi<br>Non-Saudi<br><br>Saudi<br>Non-Saudi<br><br>Saudi<br>Non-Saudi<br><br>Saudi<br>Non-Saudi<br><br>Saudi<br>Non-Saudi<br><br>Saudi<br>Non-Saudi	8	Almalki OS et al<br><br>Ennaceur S and Al-Mohaithef<br><br><br>Aedh AI et al<br><br><br><br>AlQahtani AM et al<br><br>Almuqbil M et al<br><br>Khatrawi EM and Sayed AA<br><br>Almansour A et al<br><br><br>Shati AA et al		 1339/1577 84.9<br>238/ 1577 15.1<br><br>135/167 80.8<br>32/167 19.2<br><br>88/129 68.2<br>41/129 31.8<br><br>83/86 96.5%<br>3/86 3.5%<br><br>197/291 67.7<br>94/291 32.3%<br><br>83/130 63.8<br>47/130 36.2<br><br>227/289 78.5%<br>62/289 21.5%<br><br>444/480 92.5<br>36/480 7.5	 2596/3149 82.4<br>553/3149 17.6	 2244/2558 87.7<br>314/2558 12.3<br><br>183/212 86.3<br>29/212 13.7<br><br>64/72 88.9<br>8/72 11.1<br><br>196/203 96.6<br>7/203 3.4<br><br>251/408 61.5<br>157/408 38.5<br><br>109/170 64.1<br>61/170 35.9<br><br>166/211 78.7<br>45/211 21.3<br><br>440/449 98.0<br>9/449 2.0	 3653/4283 85.3<br>630/4283 14.7	1.24 (1.09-1.40)	0.0009

**Income**	9	Almalki OS et al							
Low less than 15,000 SAR				1184/1577 75	2941/4288 68.6	1934/2558 75.6	3434/4630 74.2	1.32 (1.20-1.44)	< 0.0001
High more than 15,000 SAR				393/1577 25	1347/4288 31.4	624/2558 24.4	1196/4630 25.8		
		Ennaceur S and							
Low less than 15,000 SAR		Al-Mohaithef		152/167 91		197/212 92.9			
High more than 15,000 SAR				15/167 9		15/212 7.1			
		Aedh AI et al							
Low less than 15,000 SAR				43/86 50		40/72 55.6			
High more than 15,000 SAR				43/86 50		32/72 44.4			
		AlQahtani AM et al							
Low less than 15,000 SAR				17/67 25.4%		45/134 33.6			
High more than 15,000 SAR				50/67 74.6%		89/134 66.4			
		Almuqbil M et al							
Low less than 15,000 SAR				162/169 95.9%		241/270 89.3			
High more than 15,000 SAR				7/ 169 4.1%		29/270 10.7			
		Iqbal MS et al							
Low less than 15,000 SAR				641/675 94.9		376/397 94.7			
High more than 15,000 SAR				34/675 5.1		21/397 5.3			
		Alalmaei Asiri et al							
Low less than 15,000 SAR				178/465 38.3%		88/155 56.8			
High more than 15,000 SAR				287/465 61.7%		67/155 43.2			
		Al-khlaiwi T et al							
Low less than 15,000 SAR				207/602 34.4%%		150/383 39.2			
High more than 15,000 SAR				395/602 65.6%		233/383 60.8			
		Shati AA et al							
Low less than 15,000 SAR				357/480 74.4%		363/449 80.8			
High more than 15,000 SAR				123/480 25.6%		86/449 19.2			
**Marital status**	10	Almalki OS et al							
Married				1448/1577 91.8	3492/4209 83	2411/2558 94.3	4460/5059 88.2	1.53 (1.36-1.72)	< 0.0001
Not or single				129/1577 8.2s	717/4209 17	147/2558 5.7	599/5059 11.8		
		Ennaceur S and							
Married		Al-Mohaithef		114/167 68.3%		117/212 55.2			
Not or single				53/167 31.7%		95/212 44.8			
		Aedh AI et al							
Married				112/129 86.8		69/72 95.8			
Not or single				17/129 13.2		3/72 4.2			
		Khan YH et al							
Married				128/188 68.1		164/256 64.1			
Not or single				60/188 31.9		92/256 35.9			
Married		AlQahtani AM et al		76/86 88.4%		187/203 92.1			
Not or single				10/86 11.6%		16/203 7.9			

Married<br>Not or single		Almuqbil M et al		266/291 91.4%<br>25/291 8.6%		382/408 93.6<br>26/408 6.4			
Married<br>Not or single		Iqbal MS et al		450/537 83.8%<br>87/537 16.2%		449/535 83.9<br>86/535 16.1			
Married<br>Not or single		Alalmaei Asiri et al		244/465 52.5<br>221/465 47.5%		88/155 56.8<br>67/155 43.2			
Married<br>Not or single		Almansour A et al		258/289 89.3<br>31/289 10.7%		197/211 93.4<br>14/211 6.6			
Married<br>Not or single		Shati AA et al		396/480 82.5<br>84/480 17.5		396/449 88.2<br>53/449 11.8			
**Participants or FM working as HCP**<br>Yes<br>No	9	Ennaceur S and Al-Mohaithef		136/167 81.4<br>31/167 18.6	731/2652 27.6<br>1921/2652 72.4	181/212 85.4<br>31/212 14.6	504/2281 22.1<br>1777/2281 77.9	1.34 (1.18-1.53)	< 0.0001
Yes<br>No		Aedh AI et al		67/129 51.9<br>62/129 48.1		15/72 20.8<br>57/72 79.2			
Yes<br>No		Khan YH et al		27/188 14.4<br>161/188 85.6		66/256 25.8<br>190/256 74.2			
Yes<br>No		AlQahtani AM et al		23/86 26.7%<br>63/86 73.3%		16/203 7.9<br>187/203 92.1			
Yes<br>No		Almuqbil M et al		40/291 13.7%<br>251/291 86.3%		61/408 15.0<br>347/408 85.0			
Yes<br>No		Iqbal MS et al		228/716 31.8<br>488/716 68.2		47/356 13.2<br>309/356 86.8			
Yes<br>No		Khatrawi EM and Sayed AA		42/130 32.3<br>88/130 67.7		30/170 17.6<br>140/170 82.4			
Yes<br>No		Alalmaei Asiri et al		31/465 6.7%<br>434/465 93.3%		6/155 3.9<br>149/155 96.1			
Yes<br>No		Shati AA et al		137/480 28.5<br>343/480 71.5		82/449 18.3<br>367/449 81.7			

**Child received previous routine childhood vaccines**		Almusbah Z et al							
Yes				200/236 84.7%	391/451 86.7	300/340 88.2	523/655 79.8	1.64 (1.18- 2.29)	0.003
Vs No				36/236 15.3%	60/451 13.3	40/340 11.8	132/655 20.2		
		Aedh AI et al							
Yes	3			111/129 86%		83/112 74.1			
Vs No				18/129 14%		29/112 25.9			
Yes		AlQahtani AM et al		80/86 93%		140/203 69			
Vs No				6/86 7%		63/203 31			
**Child or Parent received influenza vac**	6	Almusbah Z et al							
Yes				100/256 39%	1823/3593 50.7	300/360 83.3	1231/4929 25		
Vs No				156/256 61%	1770/3593 49.3	60/360 16.7	3698/4929 75	3.09 (2.82- 3.39)	< 0.0001
		Almalki OS et al							
Yes				566/1577 35.9		484/2558 18.9			
Vs No				1011/1577 64.1		2074/2558 81.1			
		Al-Rasheedi AT et al							
Yes				41/198 20.7		41/341 12.0			
Vs No				157/198 79.3		300/341 88.0			
Yes		Khan YH et al		86/188 45.7		96/256 37.5			
Vs No				102/188 54.3		160/256 62.5			
Yes		Iqbal MS et al		969/1244 77.9%		275/1244 22.1%			
Vs No				275/1244 22.1%		969/1244 77.9%			
Yes		Khatrawi EM and Sayed AA		61/130 46.9		35/170 20.6			
Vs No				69/130 53.1		135/170 79.4			
**Parents received COVID-19 Vaccines**	9	Almalki OS et al							
Yes				1540/1577 97.6	3574/3666 97.5	2420/2558 94.6	4218/4586 92.0		
Vs No				37/1577 2.4	92/3666 2.5	138/2558 5.4	368/4586 8.0	3.39 (2.69- 4.28)	< 0.0001
		Al-Rasheedi AT et al							
Yes				194/198 98%		330/341 96.8			
Vs No				4/198 2%		11/341 3.2			
Yes		Aedh AI et al		129/129 100		101/112 90.2			
Vs No				0/129 0		11/112 9.8			
Yes		Khan YH et al		185/188 98.4		217/256 84.8			
Vs No				3/188 1.6		39/256 15.2			

Yes<br>Vs No		AlQahtani AM et al		85/86 98.8<br>1/86 1.2%		186/203 91.6<br>17/203 8.4			
Yes<br>Vs No		Almuqbil M et al		290/291 99.7%<br>1/291 0.3%		392/408 96.1<br>16/408 3.9			
Yes<br>Vs No		Khatrawi EM and Sayed AA		124/130 95.4<br>6/130 4.6		105/170 61.8<br>65/170 38.2			
Yes<br>Vs No		Alalmaei Asiri et al		452/465 97.2%<br>13/465 2.8%		135/155 87.1<br>20/155 12.9			
Yes<br>Vs No		Al-khlaiwi T et al		575/602 95.5%<br>27/602 4.5%%		332/383 86.7<br>51/383 13.3			
**Perceived severity: infected child or family member with COVID-19**<br>Yes<br>No	10	Almusbah Z et al		31/256 12.1%<br>225/256 87.9%	1534/4060 37.8<br>2526/4060 62.2	35/360 9.7<br>325/360 90.3	2042/4943 41.3<br>2901/4943 58.7	1.16 (1.06-1.26)	0.0007
Yes<br>No		Almalki OS et al		552/1577 35<br>1,025/1577 65		1,018/2558 39.8<br>1,540/2558 60.2			
Yes<br>No		Al-Rasheedi AT et al		34/198 17.2<br>164/198 82.8		66/341 19.4<br>275/341 80.6			
Yes<br>No		Ennaceur S and Al-Mohaithef		52/167 31.1<br>115/167 68.9		33/212 15.6<br>179/212 84.4			
Yes<br>No		Aedh Al et al		63/129 (48.8%)<br>66/129 (51.2%)		79/112 70.5<br>33/112 29.5			
Yes<br>No		AlQahtani AM et al		68/86 79.1<br>18/86 20.9		186/203 91.6<br>17/203 8.4			
Yes<br>No		Almuqbil M et al		223/291 76.6%<br>68/291 23.4%		307/408 75.2<br>101/408 24.8			
Yes<br>No		Alalmaei Asiri et al		139/465 29.9%<br>326/465 70.1%		40/155 25.8<br>115/155 74.2			
Yes<br>No		Almansour A et al				108/211 51.2			

Yes<br>No<br><br>Yes<br>No		Al-khlaiwi T et al		119/289 41.2%<br>170/289 58.8%<br><br>253/602 42%<br>349/602 58%		103/211 48.8<br><br>170/383 44.4<br>213/383 55.6			
**Perceived safety of vaccines**<br><br>Yes safe<br>Vs No (not safe)<br><br>Yes safe<br>Vs No (not safe)<br><br>Yes safe<br>Vs No (not safe)<br><br>Yes safe<br>Vs No (not safe)<br><br><br><br>Yes safe<br>Vs No (not safe)	6	Aldakhil H<br><br>Almalki OS et al<br><br>Ennaceur S and Al-Mohaithef<br><br>Aedh AI et al<br><br>Iqbal MS et al<br><br><br><br>Almansour A et al		 <br>52/56 92.9%<br>4/56 7.1%<br><br>775/1577 47.9<br>802/1577 50.2<br><br>157/167 94<br>10/167 6<br><br>50/129 (38.8%)<br>79/129 (61.2%)<br><br>731/1093 66.9%<br>362/1093 33.1%<br><br><br><br>35/289 12.1<br>254/289 87.9	 <br>1800/3311 54.4<br>1511/3311 45.6	 <br>26/70 37.1<br>44/70 62.9<br><br>443/2558 17.3<br>2115/2558 82.7<br><br>166/212 78.3<br>46/212 21.7<br><br>11/112 9.8<br>101/112 90.2<br><br>362/1093 33.1%<br>731/1093 66.9%<br><br><br><br>52/211 24.6<br>159/211 75.4	 <br>1060/4256 24.9<br>3196/4256 75.1	3.59 (3.26- 3.96)	< 0.0001
**Perceived efficacy of vaccines**<br>Yes Effective<br>Vs No (not effective)<br><br>Yes Effective<br>Vs No (not effective)<br><br>Yes Effective<br>Vs No (not effective)<br><br>Yes Effective<br>Vs No (not effective)	4	Aldakhil H<br><br>Almalki OS et al<br><br>Iqbal MS et al<br><br>Alalmaei Asiri et al		 54/56 94.4<br>2/56 3.6%<br><br>1,448/1577 91.8<br>129/1577 8.2<br><br>993/1178 84.3%<br>185/1178 15.7%<br><br>324/465 69.7%<br>141/465 30.3%	 2819/3276 86.1<br>457/3276 13.9	 41/70 58.6<br>29/70 41.4<br><br>965/2558 37.7<br>1593/2558 62.3<br><br>185/1178 15.7%<br>993/1178 84.3%<br><br>55/155 35.5<br>100/155 64.5	 1246/3961 31.5<br>2715/3961 68.5	13.44 (11.93-15.15)	< 0.0001

**Source of information about vaccines**	4	Aldakhil H							
Gov or MOH HCP				48/56 85.7%	892/2052 43.5	40/70 57.1	1201/3007 39.9		
Vs Others (Social media)				8/56 14.3%	1160/2052 56.5	30/70 42.9	1806/3007 60.1	1.16 (1.03- 1.30)	0.01
		Almalki OS et al							
Gov or MOH HCP				662/1577 41.9		983/2558 38.4			
Vs Others (Social media)				915/1577 58.1		1575/2558 61.6			
		Khatrawi EM and							
Gov or MOH HCP		Sayed AA		47/130 36.2		64/168 38.1			
Vs Others (Social media)				83/130 63.8		104/168 61.9			
		Almansour A et al							
Trusted as Gov or MOH HCP				135/289 46.7%		114/211 54.0			
Vs Others (Social media)				154/289 53.3%		97/211 46.0			
